# Leveraging the Cell Ontology to classify unseen cell types

**DOI:** 10.1038/s41467-021-25725-x

**Published:** 2021-09-21

**Authors:** Sheng Wang, Angela Oliveira Pisco, Aaron McGeever, Maria Brbic, Marinka Zitnik, Spyros Darmanis, Jure Leskovec, Jim Karkanias, Russ B. Altman

**Affiliations:** 1 Department of Bioengineering, Stanford University, Stanford, CA 94305 USA; 2 Department of Genetics, Stanford University, Stanford, CA 94305 USA; 3 Chan Zuckerberg Biohub, San Francisco, CA 94158 USA; 4 Department of Computer Science, Stanford University, Stanford, CA 94305 USA

**Keywords:** Machine learning, Network topology

## Abstract

Single cell technologies are rapidly generating large amounts of data that enables us to understand biological systems at single-cell resolution. However, joint analysis of datasets generated by independent labs remains challenging due to a lack of consistent terminology to describe cell types. Here, we present OnClass, an algorithm and accompanying software for automatically classifying cells into cell types that are part of the controlled vocabulary that forms the Cell Ontology. A key advantage of OnClass is its capability to classify cells into cell types not present in the training data because it uses the Cell Ontology graph to infer cell type relationships. Furthermore, OnClass can be used to identify marker genes for all the cell ontology categories, regardless of whether the cell types are present or absent in the training data, suggesting that OnClass goes beyond a simple annotation tool for single cell datasets, being the first algorithm capable to identify marker genes specific to all terms of the Cell Ontology and offering the possibility of refining the Cell Ontology using a data-centric approach.

## Abstract (web summary)

Classifying cells into unseen cell types remains challenging in scRNA-seq analysis. Here we show that Cell Ontology enables an accurate classification of unseen cell types through considering the cell type relationships in the Cell Ontology graph.

## Introduction

Single-cell RNA sequencing (scRNA-seq) has emerged as a powerful tool to generate comprehensive organismal atlases encompassing a wide range of organs and tissues^1–10^. One of the most important tasks in single-cell analysis is cell type annotation because all downstream analyses heavily rely on such information^11–18^. This process that aims at characterizing and labeling groups of cells according to their gene expression is currently very inefficient due to the intense need for manual curation by a panel of tissue experts for each tissue and organ. Recent efforts in scRNA-seq have produced an unprecedented large compendium of expert-curated cell type annotations, paving the way for scientists to better understand cellular diversity^3,19–22^. However, utilizing these cell type annotations is challenging due to the inconsistent terminology used to describe cell types collected by independent groups^3,4,19^. This inconsistency will likely increase as more groups generate new datasets and more cell types and states are characterized, thus substantially preventing reproducible annotations and joint analysis of multiple datasets.

A natural approach to addressing the inconsistent vocabulary challenge is to build computational methods that automatically assign cells from different datasets to categories in a controlled vocabulary. Ideally, these methods should be fully automated such that the results can be quickly updated as the ontology evolves. The Cell Ontology offers a controlled vocabulary for cell types and has been proposed as a basis for consistently annotating large-scale single-cell atlases^14,23–28^. However, computationally assigning cells to terms (i.e., cell types) in the Cell Ontology has at least two challenges. First, although the Cell Ontology contains valuable hierarchical relationships among cell types, most of these cell type terms are not associated with marker genes, which are used by experts in manual annotation and could help automate cell type annotation. Second, even though supervised learning approaches can be used to predict Cell Ontology terms that have curated annotations, they are unable to classify cells to unseen terms (i.e., terms which do not have any annotated cells in the training data). This issue is dramatically slowing research by hampering our ability to automatically annotate new datasets that pave the way to fully understanding cellular diversity as more than 95% of cell types in the Cell Ontology are unseen even in the largest datasets^19,20,29^. Collectively, these two challenges hinder progress toward comprehensive cell type annotation and cellular diversity understanding.

We developed Ontology-based single cell Classification (OnClass) to address these challenges. OnClass is able to automatically classify cells to any cell type as long as its corresponding term is captured in the Cell Ontology, even if this cell type does not have annotated cells in the training data. Throughout this paper, we refer to “unseen Cell Ontology terms” to describe cell types from the Cell Ontology that do not have any annotated cells in the training data. In contrast, we use “seen Cell Ontology terms” to denote cell types with some annotated cells in the training data. OnClass is the first method that can classify cells into specific cell types for which there are no annotated cells, rather than into a generic unassigned category as its common in previous work^11,12^. In addition, by projecting single-cell transcriptomes and the Cell Ontology into the same low-dimensional space, OnClass advances other important applications, such as robust computation of marker genes.

Here, we evaluated OnClass on nine comprehensive datasets representative of the existing biggest efforts of cell type characterization. Through careful benchmarking, we found that our method outperformed existing methods at annotating unseen Cell Ontology terms and we further demonstrated the robustness of OnClass prediction by performing cross-dataset prediction, where a substantial proportion of cell types was not part of the training set. Finally, we showed that OnClass was able to accurately identify marker genes for seen Cell Ontology terms as well as unseen Cell Ontology terms. These OnClass-computed marker genes achieved comparable performance to curated marker genes on cell type annotation, leading the way for creating an organism-wide molecular representation of cellular diversity.

## Results

### Overview of OnClass

**Fig. 1:**
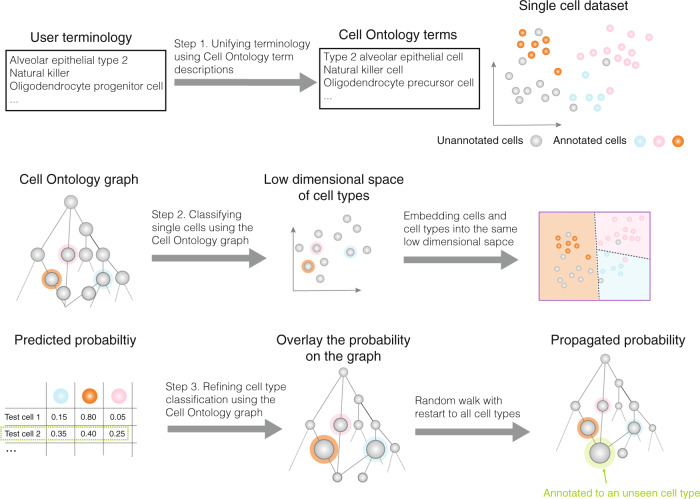
OnClass overview. OnClass first maps user terminology to Cell Ontology terms based on the text embedding similarity. It then embeds cell ontology terms into a low-dimensional space using the Cell Ontology graph. Single-cell transcriptomes are then projected into the same low-dimensional space using a nonlinear transformation. Finally, OnClass refines the annotation of each cell by first overlaying confidence scores on the Cell Ontology graph and then propagating these scores using random walk with restart.

The Cell Ontology is a controlled vocabulary that organizes 2331 cell types anatomically derived into a hierarchy based on the “is_a” relation^25^. Each cell type is associated with a text description in the Cell Ontology. OnClass uses both the Cell Ontology graph and the cell type description to classify single cells (see “Methods”). OnClass has three steps. In the first step, we map the user terminology to Cell Ontology terms based on the text embedding similarity using natural language processing (NLP)^30^. Then, in the second step, we embed cell types into a low-dimensional space using the Cell Ontology graph^31,32^ (Supplementary Note 1). Single-cell transcriptomes are then projected into the same low-dimensional space by finding a nonlinear transformation that projects each annotated cell to the region of its cell type. Lastly, in the third step, we refine the annotation of each cell by first overlaying confidence scores on the Cell Ontology graph and then propagating these scores using random walk with restart. Such a framework enables us to classify cells to unseen Cell Ontology terms based on their distances to other seen terms on the Cell Ontology graph (Fig. 1). OnClass is a Python-based open source package able to compute cell type similarities between the hierarchical structure of existing cell ontologies, such as the Cell Ontology^25^ and the Allen Ontology^20,33^. Moreover, we provide a pre-trained model, which given an input gene expression matrix can predict cell types for millions of cells in a few minutes on a modern server. The fully tunable version is also available to the users and our implementation can take the input gene expression matrix in a wide range of formats.

### Cell Ontology reflects cell similarity

We first verified the merit of our approach by comparing three types of cell type similarities: Cell Ontology graph-based similarity (cosine similarity between the random walk with restart distributions), cell type text description-based similarity (cosine similarity between low-dimensional text representations of cell type description), and gene expression-based similarity (“Methods”). We observed that Cell Ontology graph-based similarity was strongly correlated with cell type text description-based similarity. For example, the average text description-based similarity of direct neighbors on the Cell Ontology graph was 0.67, which was far higher than for cell types that are four-hop away (similarity equals to 0.49; Supplementary Fig. 1a). We further found that sibling terms (i.e., terms that have the same parent node) are more similar to each other in terms of text-based cell type similarity with the increase of depth on the Cell Ontology graph, reflecting OnClass’ ability to model the similarity among cell types at different granularity (Supplementary Fig. 1b). We also observed that cell types with similar text description tend to have similar gene expression profiles (Supplementary Fig. 1c). We, therefore, used the text description-based similarity to augment edges on the Cell Ontology graph. The text description-based similarity was also used to help users map curated free text annotation to the Cell Ontology (Fig. 1, Supplementary Data 1).

Next, we examined whether cell types that were deemed similar by the Cell Ontology would have similar gene expression profiles. Using a collection of annotated cells as the benchmark, we observed strong correlations between these two types of similarities. For instance, the correlation between the gene expression-based similarity and the Cell Ontology graph-based similarity was 0.65 (*p*-value < 1e−7) in lung and 0.93 (*p*-value < 1e−15) in pancreas (Supplementary Figs. 2, 3). The strong correlation between these two types of similarities suggested that the Cell Ontology graph follows the guilt-by-association principle^34^. In particular, the guilt-by-association principle states that nearby nodes (Cell Ontology graph-based similarity) have similar features (gene expression-based similarity). According to this principle, one might be able to use the Cell Ontology graph to transfer annotations from seen cell types to any unseen cell types, which serves as the basis of OnClass. OnClass’ ability to annotate cells with any cell type in the Cell Ontology motivates us to examine whether we could improve cell type annotation on large and diverse collections of scRNA-seq datasets.

### Improved unseen cell type annotations within the same dataset

**Fig. 2:**
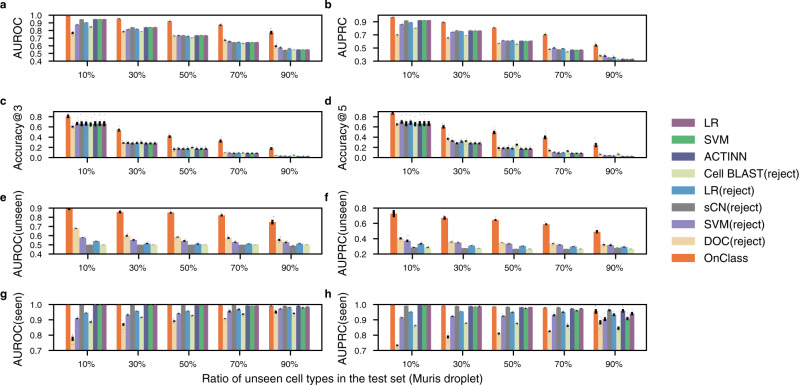
Performance of OnClass on unseen cell type annotation. **a**–**h** Bar plots comparing OnClass and existing methods in terms of AUROC (**a**), AUPRC (**b**), Accuracy@3 (**c**), Accuracy@5 (**d**), AUROC on unseen cell types (**e**), AUPRC on unseen cell types (**f**), AUROC on seen cell types (**g**), and AUPRC on seen cell types (**h**) in Muris droplets. *x*-axis shows the proportion of unseen cell types in the test data. Error bar represents standard errors across 5 replicates. Mean is used to measure the centre for the error bar.

To investigate how well OnClass could annotate unseen Cell Ontology terms, we used datasets 1–6 (“Methods”) and split the cells from each dataset into test and training in a controlled fashion accounting for different proportions of unseen Cell Ontology terms in the test set. All the reported unseen scores are calculated by averaging scores from individual unseen cell types. Overall, we observed that OnClass outperformed existing approaches (Fig. 2, Supplementary Figs. 4–10). We first compared OnClass with existing single cell classification methods that could not reject a cell to all seen cell types, including ACTINN, SVM, and LR. We found that OnClass substantially outperformed these approaches on all metrics across different datasets. For instance, when 70% of cell types were not seen in the training set, all the other approaches yielded an AUROC less than 0.67 in Muris droplet, which was substantially lower than 0.87 for OnClass. We achieved similar conclusions based on the binary classification metrics Accuracy@3 and Accuracy@5. Alltogether, we found that the OnClass capability of accurately annotating cell types, even when the dataset has increasing proportions of unseen cell types, opens a new avenue for automated cell type annotations. Existing top-performing methods^15^ cannot annotate cell types not present in the training dataset, inevitably limiting their usability to aid the cell biology community identifying novel cell types across single-cell transcriptomic datasets. Notably, even though Tabula Muris Senis is one of the most diverse collections of annotated single-cell transcriptomics profiles, it still only covers less than 5% of all cell types described in the Cell Ontology.

**Fig. 3:**
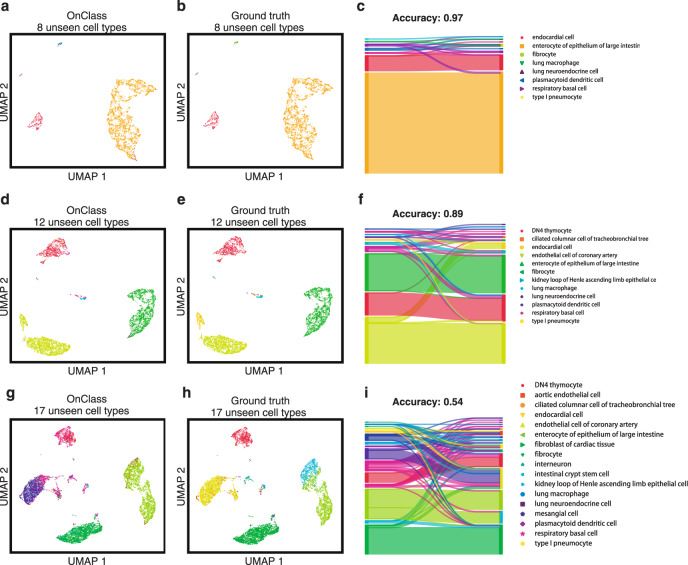
OnClass can accurately assign cell types that are not present in the training set. **a**, **b**, **d**, **e**, **g**, **h** 2-D UMAP plots showing the predicted Cell Ontology terms of OnClass (**a**, **d**, **g**) and ground truth labels (**b**, **e**, **h**) for 8 (**a**, **b**), 12 (**d**, **e**), and 17 (**g**, **h**) unseen Cell Ontology terms in Muris FACS. The presence of the same color between OnClass predicted labels and ground truth labels means correct annotation. **c**, **f**, **i**, Sankey diagrams of the resulting mapping between predicted labels (left) to ground truth labels (right) for 8 (**c**), 12 (**f**), and 17 (**i**) unseen Cell Ontology terms.

We next compared OnClass with methods that can reject cells to all seen cell types, including sCN(reject), LR(reject), SVM(reject), Cell BLAST(reject), and DOC(reject). However, these methods can only group the rejected cells into a generic “unknown” type. To the best of our knowledge, OnClass is actually the first method that can classify cells into specific unseen cell types. To enable better comparisons between OnClass and these algorithms, we decided to extend such approaches by classifying “unknown” cells to the nearest cell type in the Cell Ontology (see “Methods”). We observed significant improvement of OnClass in comparison to these methods (Fig. 2, Supplementary Figs. 4–10). For example, when 30% of Cell Ontology terms were unseen in the training data, OnClass obtained 0.56 Accuracy@3 and 0.60 Accuracy@5 in Muris droplet, which was at least 50% better than any existing approaches (Fig. 2c, d). On a randomly selected set of 8 unseen terms in Muris FACS, OnClass was able to accurately classify 97% of cells (Fig. 3a–c). When challenging the method on larger sets of 12 and 21 unseen terms, OnClass still accurately classified 89 and 54% of cells, respectively (Fig. 3d–i). In contrast, all existing methods could only make random predictions in this challenging setting.

Next we investigated how the performance on unseen cell types was related to the number of seen cell types within the 2-hop region (shortest distance smaller than 3 on the Cell Ontology graph) (Supplementary Fig. 11a, b), their distance to the nearest seen cell type and number of annotated cells (Supplementary Fig. 11c, d), and sample sizes (Supplementary Fig. 12). By combining diverse datasets into a single training set for OnClass, we observed that the performance of OnClass was better for unseen cell types who are surrounded by more seen cell types (Supplementary Fig. 11a, b) and have smaller distances to the nearest seen cell type (Supplementary Fig. 11c, d). Moreover, we also observed the desirable performance of OnClass when evaluating the overall accuracy for cell types belonging to a certain tissue (Supplementary Figs. 13–18).

### Cross-dataset unseen cell type annotation

**Fig. 4:**
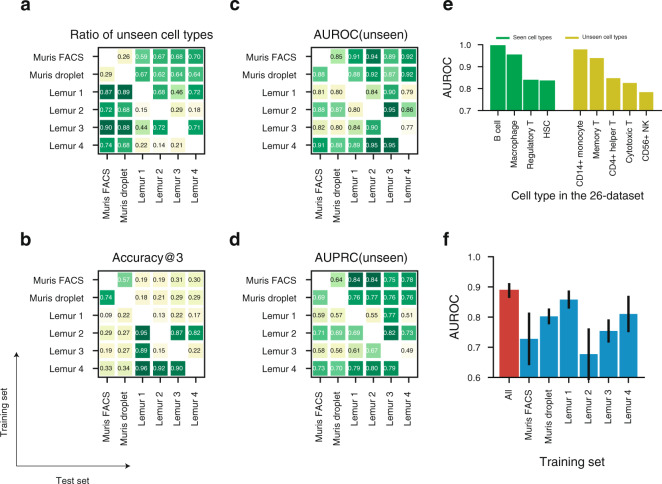
Training with different datasets and proportions of unseen cell types highlights OnClass versatility and accuracy. **a**–**d** Heatmaps showing the ratio of unseen cell types (**a**), Accuracy@3 (**b**), unseen AUROC (**c**), and unseen AUPRC (**d**) in cross-dataset prediction. The *x*-axis is the test set and the *y*-axis is the training set. **e** Bar plot showing the AUROC of OnClass on 9 cell types, including 4 present in the training set (green) and 5 not present in the training set (yellow). **f** Bar plot showing the AUROC of OnClass using all six datasets (red) and each individual dataset (blue) as the training set to classify cells in the 26-dataset. Error bar represents standard errors. Mean is used to measure the centre for the error bar.

We next examined the robustness and applicability of OnClass by using it to annotate diverse datasets across species, animals, technologies, and organs. In particular, we trained OnClass on cells from one dataset (training set) and then used it to classify cells from another dataset (test set). Interestingly, we observed a large proportion of unseen cell types across the different datasets we have collected, even when they are from the same species (e.g., 72% of cell types in Lemur 1 are not in Lemur 4) (Fig. 4a). Even in the presence of such disparate sets of cell types, OnClass was still able to achieve good prediction performance across the datasets in comparison to comparison approaches (Fig. 4b–d, Supplementary Fig. 19, Supplementary Data 2). As an example, OnClass obtained 0.79 AUPRC when trained on Lemur 4 and tested on Lemur 1. On a more challenging setting of 67% unseen cell types between two different species (Muris FACS and Lemur 2), OnClass still obtained 0.94 AUROC on unseen cell types. The cross-dataset evaluation supports OnClass as a robust method for automated cell type classification in datasets with large numbers of unseen cell types.

Building on this, we build a training model using all these 6 datasets. We then evaluated the ability of this training set to predict cell types in the 26-dataset^35^, a dataset independently generated and annotated which contains a diverse set of 105,476 cells collected from 26 single-cell datasets (26-dataset) representing 9 technologies and 11 studies (see “Methods”). We observed an average AUROC of 0.90 for seen cell types and 0.87 for unseen cell types (Fig. 4e). Notably, the performance of OnClass trained on all 6 diverse datasets was substantially better than trained on each individual dataset (Fig. 4f). This improvement indicates that OnClass performance can be easily improved by adding newly annotated datasets to the training, even if they are from different tissues, technologies or even species.

**Fig. 5:**
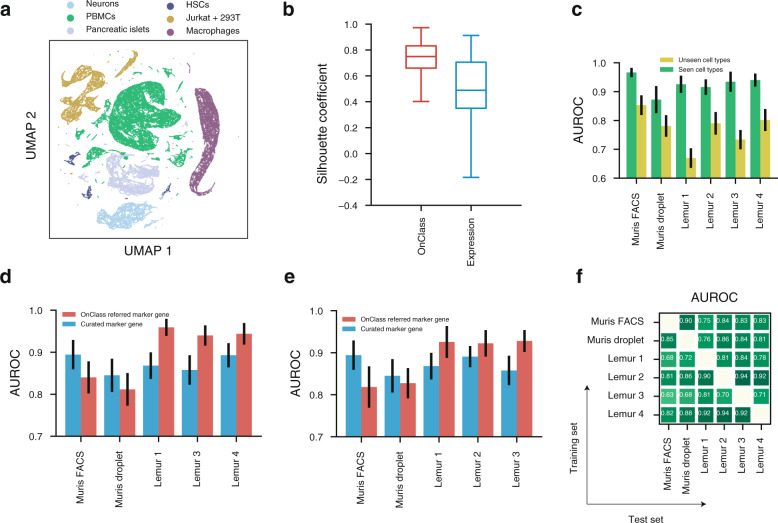
OnClass can assign marker genes to seen and unseen cell types. **a** 2-D UMAP plot showing OnClass’s integration of 26 datasets^35^ on 6 groups. We combined the 9 cell types (list here) into six groups (Neurons, PBMCs, Pancreatic islets, HSCs, Jurkat+293T, and Macrophages). **b** Box plot showing the comparison between OnClass and expression on data integration in terms of the silhouette coefficient (*P*-value < 7e−293). *P*-value is determined using a two-sided *t*-test (*n* = 6 cell types). Minima, maxima, centre, bounds of box, and whiskers represent quantile 1–1.5*interquartile range (IQR), quantile 3+1.5*IQR, median, quantile 1, and quantile 3. **c** Bar plot showing the AUROC of predicting marker genes using different datasets. Error bar represents standard errors across 52 unseen cell types and 17 seen cell types for Muris FACS, 48 unseen cell types and 21 seen cell types for Muris droplet, 55 unseen cell types and 13 seen cell types for Lemur 1, 46 unseen cell types and 22 seen cell types for Lemur 2, 55 unseen cell types and 13 seen cell types for Lemur 3, and 46 unseen cell types and 22 seen cell types for Lemur 4. Mean is used to measure the centre for the error bar. **d** Bar plot comparing the AUROC of using OnClass-computed marker genes and curated marker genes to classify cells in different datasets using marker genes obtained from Lemur 2 (**d**) and Lemur 4 (**e**). Error bar represents standard errors of *n* = 17, 21, 13, 22, 13, 22 for Muris FACS, Muris droplet, Lemur 1, Lemur 2, Lemur 3, Lemur 4, respectively. Mean is used to measure the centre for the error bar. **f** Heatmap showing the AUROC of using marker genes to classify cells in the cross-dataset setting. The *x*-axis is the test set and the *y*-axis is the training set.

Furthermore, the predicted cell type annotations can be used as features to cluster and integrate cells from different datasets. We used the predicted cell type annotations to integrate these 26 datasets following the same procedure as the previous work^35^. We observed good performance by using OnClass, where cells were clustered based on cell types rather than artifacts related to platforms (Fig. 5a). We further quantified the integration performance using the silhouette coefficient^36^ and observed a significant improvement in comparison to expression-based integration using Scanorama^35^ (*P*-value < 7e−293 using a two-sided test) (Fig. 5b), indicating once again OnClass’s robustness to annotating cells from different batches and across datasets.

### Identifying marker genes for unseen Cell Ontology terms

Given the accurate annotation of both seen and unseen Cell Ontology terms, we were then interested in using OnClass to identify marker genes for all the existing Cell Ontology terms. Marker genes are the key to expert curation but the existing knowledge is incomplete and limited to extensively studied cell types. We started by using OnClass to identify marker genes for both seen and unseen Cell Ontology terms across the six datasets we used for the training set (Fig. 5c). OnClass was able to identify the correct marker genes for seen Cell Ontology terms with AUROCs ranging from 0.87 to 0.97 on these six diverse datasets. More importantly, since OnClass does not rely on the transcriptome profiles to identify marker genes, it was able to find marker genes for unseen Cell Ontology terms as well. For example, OnClass obtained an AUROC 0.87 for unseen Cell Ontology terms in Muris FACS and an AUROC 0.82 for unseen Cell Ontology terms in Muris droplet. We incorporated these OnClass-computed marker genes into our provisional Cell Ontology (Supplementary Data 3), in the hope of facilitating future expert curation. This data is easily accessible through our portal (http://onclass.ds.czbiohub.org) and although these marker genes are by no means a completely accurate representation of cell type features, they are the first attempt at creating a comprehensive knowledge base of marker genes representative of the entire cellular diversity.

Finally, we sought to examine whether OnClass-computed marker genes could be used to accurately annotate cells. We performed a cross-dataset validation, where cells in one dataset were used to find marker genes (training set) and cells from another data were then classified using these marker genes (test set). We found that the performance of using OnClass-computed marker genes was comparable to curated marker genes (Fig. 5d, e, Supplementary Fig. 20). More importantly, for those Cell Ontology terms that have no curated marker genes, OnClass-computed marker genes also achieved accurate cell type annotation performance, especially for datasets from the same species (Fig. 5f). Of note, because the performance of OnClass depends on the existence of good quality cell annotations associated with single-cell transcriptomics profiles (Fig. 4f), as more data becomes available, we anticipate substantial improvement on the identification of robust and accurate marker gene sets.

## Discussion

Cell type annotation is a key step of the single-cell transcriptomics workflows because all the subsequent analysis depends on the assigned labels. It is also a bottleneck and therefore an active area of research, with most computational methods focusing on classifying cells into either existing labels or as a generic unseen cell type^11,12^. Despite encouraging results based on these approaches, these methods fail to provide meaningful information on cell types that have not yet been molecularly characterized. As more cell types are discovered, there is an increased need to go beyond what is available in the different training sets of previously characterized cell types. Our method takes an important step forward by expanding the set of possible cell types to the whole Cell Ontology and paving the way toward automating the process of cell type annotation with accuracy even for previously unseen Cell Ontology terms. In contrast to existing efforts that utilize the Cell Ontology^14,28,37,38^ or other ontologies, such as the Disease Ontology^39^, to improve the classification task, our method is able to classify cells into any cell type within the Cell Ontology, even if such cell type does not have annotated cells in the training set.

To make OnClass even more applicable to single cell datasets, a natural follow up is to automate the process of mapping free text annotations from different datasets to the Cell Ontology. Here we already offer an NLP model that is able to map free text annotations to Cell Ontology terms and we plan to further improve the NLP models to consider not only text data but also the Cell Ontology graph structure. There are also other exciting efforts toward unifying the taxonomy of neural cell types^40^ and the goal is to extend the NLP models in such a way that we will integrate with such efforts and provide accurate mapping from free text annotation to the Cell Ontology. We would also like to develop methods to correct the batch effects with the existence of a large number of unseen cell types. Existing batch correction methods exploit the idea of finding mutual nearest neighbors between two datasets^41^, which could be substantially challenging when most of the cell types are only seen in one dataset. Batch correction between these datasets might further improve the performance of OnClass.

The proposed three-step framework enables OnClass to be integrated with any existing cell type classification method and/or batch correction. In particular, users can skip the second step and provide OnClass the predicted probability scores of seen Cell Ontology terms as the input to the third step. Such probability scores can be obtained by any off-the-shelf cell type annotation approach and OnClass will then perform the third step to extend these scores to unseen cell types based on the Cell Ontology graph. Since the user-selected approach does not need the ability to classify cells into unseen cell types nor reject cells to all seen cell types, users can thus select their preferred approach based on computational time, memory usage and model calibration ability. For example, using logistic regression as the base classifier enables users to also obtain well-calibrated OnClass predictions^42^. OnClass is also able to incorporate advanced single cell representations, such as MARS^43^, by using them instead of the gene expression profile as the input.

One current limitation of using the Cell Ontology is that it was not developed specifically for scRNA-seq and, therefore, likely misses cell types and cell states. Although this problem is beyond the scope of OnClass, an interesting avenue of research is to pursue such populations by investigating the probability scores predicted by OnClass. In particular, we found that OnClass’ uncertainty in classifying cell types was higher for cell type neighbors that have very close probability scores across all cells. To address this, we propose to extend the current ontology by “inserting” a new node between the two cell types and define it as a new cell population, which was neither seen in the training set nor documented in the Cell Ontology. Some preliminary results are shown in Supplementary Table 1 and we plan to thoroughly investigate these with the goal of building a data-driven Ontology for individual species. Another limitation of OnClass is that it cannot distinguish topologically identical unseen cell types, such as siblings with the same parents in the Cell Ontology graph. Although the above text-based approach enables us to avoid random guesses, OnClass might always assign the cell to the most weighted neighbors when there are multiple topologically identical neighbors. We found that 10.6% of cell type sibling pairs are indistinguishable in Cell Ontology. However, with the growing annotation of new cell types, more cell types will be seen in the training set and we hope this limitation will be circumvented by the presence of more annotated datasets. As a proof-of-concept, we compared the performance by using 6 datasets to only using individual dataset as the training set and observed that by using all 6 datasets, OnClass obtained substantially improved performance on cell type classification (Fig. 4f).

In sum, OnClass is a robust, accurate, efficient and reproducible solution to the problem of cell type classification in single cell RNA sequencing experiments. The algorithm is implemented in Python (https://github.com/wangshenguiuc/OnClass) and it has been included in PyPy to facilitate its integration with current workflows. The marker genes database for mouse is available as an online web server (http://onclass.ds.czbiohub.org/) and as part of the package we provided a pre-trained model that can output cell type annotations for millions of cells in a few minutes on a modern server. By leveraging the structure of the cell ontology, OnClass pushes the boundaries of automated cell classification, enabling cell annotation into categories never seen in the training dataset, letting researchers take advantage of large references such as Tabula Muris Senis, the Allen Brain Atlas and the upcoming Human Cell Atlas^44^.

## Methods

### scRNA-seq datasets

We used the following datasets:

*Datasets 1 & 2*: Tabula Muris Senis^19^ FACS (smartseq2) and droplet (10×);

*Datasets 3–6*: Tabula Microcebus Lemur 1–4 (10×). The data is available on Figshare (https://figshare.com/projects/Tabula_Microcebus/112227).

*Dataset 7*: The Allen Brain Atlas^45^.

*Dataset 8:* Krasnow Lung Atlas (10X)^22^.

*Dataset 9:* A collection of 26 datasets from Scanorama^35^.

A summary description of all these datasets is shown in Supplementary Table 2. Cell type annotations present in Tabula Muris Senis and Tabula Microcebus were manually mapped to the Cell Ontology vocabulary. Cell type annotations present in the Allen Brain Atlas were manually mapped to the Allen Ontology. We manually mapped cell types in the 26-dataset to Cell Ontology terms (Supplementary Table 3), since these datasets did not provide cell type annotations that were mapped to the Cell Ontology vocabulary. In the 26-dataset, PBMC was annotated as peripheral blood mononuclear cell (CL:2000001). This cell type is not part of Tabula Muris Senis (because blood is not on the tissues in the atlas), but many of its descendants were in TMS. As a result, we excluded this cell type in our analysis. After the mapping, there were 9 different Cell Ontology terms in these 26 datasets. We denoted these datasets as 26-dataset in this paper. The nearest seen Cell Ontology terms and the nearby cell types for each of these 9 cell types are shown in Supplementary Table 4 and Supplementary Figs. 21–22. We also performed a leave-one-cell-type-out evaluation using all cell types from Tabula Muris FACS. We used one cell type as the unseen cell type and the remaining cell types as the seen cell types in each leave-one-cell-type-out set. To create the training and test set, we first randomly split the cells of the seen cell types into 95% training cells and 5% test cells. We then combined the 5% test cells of these seen cell types with all cells of the unseen cell type as the test set. The 95% training cells were used as the training set.

### The Cell Ontology

We downloaded the Cell Ontology from The OBO Foundry (http://www.obofoundry.org/ontology/cl.html)^24^. We used the “is_a” relation in the Cell Ontology to construct an undirected graph of cell types. There were in total 2331 nodes in the constructed graph, corresponding to 2331 different cell types. The 2331 cell types in the Cell Ontology were not species-specific and cell states were not included.

### The Allen Ontology

We downloaded a Cell Type Taxonomy from the Allen Brain Atlas project, which included 289 cell types (https://transcriptomic-viewer-downloads.s3-us-west-2.amazonaws.com/mouse/dendrogram.zip)^46^. There were in total 289 cell types and all edges in this graph had the same weight. This Cell Type Taxonomy is referred to as the Allen Ontology in this paper.

### Cell type text description similarity

The text description of each cell type was obtained from the Cell Ontology^25^ using the field “def:”. Each cell type was associated with a sentence describing this cell type. For example, the description of T cell is “*A type of lymphocyte whose defining characteristic is the expression of a T cell receptor complex*.” If the sentence was missing for a given cell type, we used the cell type name as the description. We then used NLP techniques to calculate text-based cell type similarity between two text descriptions. In particular, we jointly embedded sentences of all cell types using an off-the-shelf text embedding method^30^. We then obtained the text-based cell type similarity using cosine similarity between these embeddings. This text-based cell type similarity was used as the edge weight of the Cell Ontology. Because the Cell Ontology might contain missing edges, we further complete this Cell Ontology using text-based cell type similarity. We added a link between two cell types on the Cell Ontology graph if there distance is smaller than ${d}_{c}$ and their text-based similarity is larger than 0.8. To generate more robust cell type graph, we created three such graphs by setting ${d}_{c}$ to 2, 3, and 4, respectively (${d}_{c}=2$ means no new edge is added). We then train one model based on each graph and integrate all models through averaging their predicted scores. Since the Allen Ontology does not contain cell type text definition, we still kept weights of all edges in the Allen Ontology as 1.

Although the Cell Ontology provides controlled vocabulary for cell type annotation, there could be datasets that were annotated to cell types using other vocabulary by experts. We referred to cell types annotated with other vocabulary as user terminology (Fig. 1a). For example, T cell in the Cell Ontology could have synonyms such as T-cell and T lymphocyte. Asking users to manually map their vocabulary to the Cell Ontology vocabulary is extremely time-consuming, error-prone and requires domain expertise. We used natural language processing (NLP) techniques to automate this process. In order to map free text annotations to the controlled vocabulary in the Cell Ontology, we first used the same text embedding method^30^ to embed free text annotations and cell ontology terms into the same low-dimensional space and then found the nearest cell ontology term for each free text annotation. Although the nearest Cell Ontology term will be deemed as the corresponding terminology in the Cell Ontology, we still provided users a shortlist of top ranked terms to prevent potential noise in text description.

We further refined the NLP-based mapping using a maximum weighted bipartite graph matching algorithm. We first constructed a bipartite graph, where source nodes are user-provided free annotation and target nodes are Cell Ontology terminologies. The weight of an edge between a source node and a target node was determined by the NLP-based similarity. We then found the maximum weighted matching between source nodes and target nodes using python package networkx^47^. This matching guarantees that each user-provided free annotation is matched to at most one Cell Ontology terminology and each Cell Ontology terminology is also matched to at most one user-provided free annotation.

### Embedding the Cell Ontology into the low-dimensional space

OnClass computed a compressed, low-dimensional representation of each cell type based on the constructed cell type graph. We used clusDCA^31,32^, which had been proposed to embed the Gene Ontology, to embed the Cell Ontology. clusDCA first computes a propagated cell type graph by applying the random walk with restart^48,49^ to the cell type graph. It then obtains the low-dimensional representation of each cell type by using the singular value decomposition (SVD)^50^ to reduce the dimensionality of this propagated cell type graph. To make our method more robust, we set the restart probability of the random walk with restart to 0.5, 0.6, 0.7, and 0.8 to obtain four different diffusion graphs respectively. We trained one model based on each diffusion graph and then averaged predictions of four different models to obtain the final prediction. Moreover, we only embedded seen cell types into the low-dimensional space by exclusively selecting rows and columns of seen cell types in the diffusion graph. This could substantially reduce running time and produce more compact representation. A detailed description of embedding cell types can be found in Supplement (Supplementary Fig. 1, Supplementary Note).

### Cell type annotation

OnClass used a bilinear neural network model to predict the Cell Ontology term for a novel cell. Let *M* be an *m* by *n* matrix of input gene expression data, where *m* was the number of cells and *n* was the number of genes. Let *Y* be an m by *c* label matrix, where *c* is the total number of Cell Ontology terms in the Cell Ontology. *Y*_*ij*_ *=* 1 if cell *i* is annotated to Cell Ontology term *j*, otherwise *Y*_*ij*_ *=* 0. Note that *c* is often much larger than the number of seen Cell Ontology terms in the training data, as the majority of Cell Ontology terms are unseen in the training data. The corresponding columns of unseen Cell Ontology terms are all zeros in the label matrix. Let *E* be a *c* by *q* matrix of the low-dimensional representations of cell types, where *q* is the dimension of cell type embedding dimensionality. *E* is the output of clusDCA and it is fixed during optimization. We further concatenated a diagonal matrix to *E*, resulting in a *c* by *q* *+* *c* matrix *X*, where the first *q* columns are the embedding of the Cell Ontology graph and the last c columns are a diagonal matrix. This diagonal matrix has the same effect as bias terms in conventional machine learning models to ease the optimization process. OnClass optimized the following cross-entropy loss:

1 $$L={\it{\Sigma}}_{i=1}^{m}{\it{\Sigma}}_{j=1}^{c}{Y}_{ij}log(exp(Relu(Relu({M}_{i}{W}_{1}){W}_{2}){X}_{j}^{T})/{\it{\Sigma }}_{k=1}^{c}exp(Relu(Relu({M}_{i}{W}_{1}){W}_{2}){X}_{k}^{T}){)}$$

where $W_1 \in R^{n \times h}$ and $W_2 \in R^{h \times (q+c)}$ were the parameters that needed to be estimated. $Relu$ was the rectifier function for nonlinear transformation^51^. *h* was the number of hidden dimensions and set to 5 with the consideration of potentially small numbers of seen cell types in the real world dataset. OnClass used ADAM^52^ to optimize this objective function.

After the optimization, the Cell Ontology term of a new cell with expression vector *z* could then be predicted as:

2 $${p}_{j}=exp\big(exp(Relu(Relu(z{W}_{1}){W}_{2}){X}_{j}^{T})/{\it{\Sigma }}_{k=1}^{c}exp\big(exp(Relu(Relu({{{{{\boldsymbol{z}}}}}}{W}_{1}){W}_{2}){X}_{k}^{T})$$

where *p*_*j*_ was the probability that this cell belonged to Cell Ontology term *j*. $P=\{{p}_{1},\,{p}_{2},...,{p}_{c}\}$ was the probability distribution that this cell belonged to each Cell Ontology term, including both seen Cell Ontology terms and unseen Cell Ontology terms. As a result, OnClass could automatically assign cells to any term in the Cell Ontology, even if it does not have any annotated cells in the training data.

OnClass allows users to input overlapping cell types. For example, when users input two cell types T cells and CD4+ T Cells, OnClass could annotate a test cell to either a T cell or a CD4+ T cell. OnClass software also lets users decide whether to include only the most fine-grained terms or not through using the exclude_non_leaf_ontology hyperparameter in OnClass software.

### Calculating cell type similarity

We calculated three types of cell type similarities: the Cell Ontology graph-based similarity (Ontology-based similarity), the text description-based similarity (text-based similarity) and the gene expression-based similarity. The Ontology-based similarity is the cosine similarity between the random walk with restart equilibrium distribution of two cell types. The text description-based similarity is the cosine similarity between low-dimensional text representations of cell type description. We also used the text description-based similarity to map free text annotations to Cell Ontology controlled vocabulary. To calculate gene expression-based similarity, we first followed McImpute^53^, a low-rank matrix completion based imputation method, to impute missing values before calculating the cell type similarity. The similarity of cells are calculated using the low-dimensional vector instead of the original gene expression vector. We used the gene expression of all FACS cells in TMS to calculate the gene expression-based similarity. The calculation was performed per organ.

### Evaluation of cell type annotation

We evaluated across different proportions of seen Cell Ontology terms in the test set ranging from 10 to 90%, where 10% indicates that 10% of Cell Ontology terms in the test set have no annotated cells in the training data. For a proportion *k* percentage, we first randomly selected *k* percentage of Cell Ontology terms as seen Cell Ontology terms and remaining Cell Ontology terms as unseen Cell Ontology terms. All cells belonging to these unseen Cell Ontology terms were used as the test set. For the seen cell types, we randomly split their cells into five equal size folds, where one-fold was used as the training set and the remaining four-folds were used as the test set. We created a five-fold of test and training here according to the initial annotation process in Tabula Muris Senis, where about 20% of cells (3-month mice) were annotated first and then extended to the remaining 80%. The test data thus contained all cells in each of the unseen Cell Ontology terms and 80% of cells in each seen Cell Ontology terms. We performed cross-validation by repeating this procedure 5 times for each proportion.

To reflect the biological input we got from the tissue experts of the Tabula Muris consortium, we purposely decided to only consider a cell type if none of its descendants was in the training set or the test set. For example, for the case when lymphocyte cells and T cells are both in the dataset, we are going to exclude lymphocyte cells but include T cells in our dataset. Such a selection process enables all cell types to be mutually exclusive, thus avoiding information leakage, while at the same time provides the biology community with a set of labels more representative of the current knowledge in the field. We evaluated our method and comparison approaches on four metrics, including the area under the receiver operating characteristic curve (AUROC), Accuracy@3, Accuracy@5, and the area under the precision recall curve (AUPRC). As we were evaluating a large number of classes (i.e., more than 80 cell types), it was important to address the bias from class imbalance during evaluation. Therefore, we used the macro-average AUROC rather than the micro-average AUROC to summarize results across different Cell Ontology terms. Macro-average AUROC calculates the areas under the curves for each class independently and then takes the average. Likewise, we also used the macro-average AUPRC. To better understand the performance of OnClass, we randomly sampled 3 times as many negative samples as positive samples, making the random AURPC 0.25 for all comparison. AUROC(useen) and AUPRC(unseen) are such scores only averaged across unseen cell types. Accuracy@3 and Accuracy@5 are widely used ranking metrics, which assesses the correctness of the top 3 or 5 predicted Cell Ontology terms, respectively. A prediction would be deemed as correct if any of the top 3 (5 for Accuracy@5) predicted Cell Ontology terms is the correct Cell Ontology term. When calculating these six metrics, we only considered cell types that appeared in either the training set or the test set. To evaluate the performance per organ, we used a fixed proportion of 50% of unseen cell types in the test set.

### Comparison approaches

We compared our method with eight existing methods: ACTINN, singleCellNet (sCN(reject)), one-vs-rest logistic regression (LR), one-vs-rest logistic regression with rejection (LR(reject)), multi-class SVM, multi-class SVM with rejection (SVM(reject)), Cell BLAST(reject) and DOC(reject). These approaches represent prevalent classification methods based on random forest (singleCellNet), neural network (ACTINN), logistic regression, and support vector machine. Our choice of methods was based on a recent benchmarking publication^15^. These seven methods were divided into two groups based on whether this method can reject a test cell to all seen cell types.

ACTINN, SVM, and LR cannot reject a cell to all seen cell types. ACTINN used a three-layer neural network to predict the cell type^13^. We used the implementation of ACTINN from the authors (https://github.com/mafeiyang/ACTINN) and ran it on TMS. We used the default parameters for ACTINN since these parameters were used in their paper to annotate cells in the Tabula Muris^3^, an earlier version and subset of our dataset. LR and SVM was the standard machine learning classifier for multi-class classification on large-scale datasets. We used the one-vs-rest logistic regression instead of the multinomial logistic regression. For these three methods that cannot reject a cell into a new cell type, the probability of assigning a cell to any unseen cell type will be zero.

sCN(reject), LR(reject), DOC(reject), SVM(reject), Cell BLAST(reject) can reject a cell to all seen cell types. sCN(reject) used gene pairs as features and random forest as the classifier to predict the cell type^11^. Notably, sCN was able to classify cells into an unknown cell type. We obtained the implementation of singleCellNet from (https://github.com/pcahan1/singleCellNet). Recent work reported that sCN was not scaled to large datasets^15^. We further noted that sCN was not able to cross-validate rare cell types with less than 50 cells. We reimplemented part of sCN to enable its annotation for rare cell types and made the code available as part of our package. To make it scalable to TMS, we ran it on the dimensionality reduced gene expression matrix instead of the original gene expression matrix and reduced the number of trees in sCN to 20. DOC(reject) is an advanced machine learning method for classifying unseen text documents, which was a natural solution to our problem and could be directly applied here^54^. The key idea of DOC(reject) was to find a data-driven probability cutoff for the unknown class rather than using a fixed probability cutoff of 0.7 as LR did. However, DOC(reject) was also not able to classify cells into the specific cell type. As the original DOC(reject) codebase was developed for word sequences classification and could not directly take gene expression as input, we reimplemented and replaced its underlying convolutional neural network classifier with a multinomial logistic regression. LR(reject) and SVM(reject) were based on the standard classification methods logistic regression and support vector machine. The key difference here is that a cell will be rejected to all seen cell types if its probability to the most likely cell type is less than 0.7. The probability cutoff 0.7 was used in a previous large-scale single cell annotation pipeline^15^. We also calibrated the output score of SVM as previous work did^15^. Cell BLAST(reject)^37^ was a recently published work that used the Cell Ontology graph to propagate predicted scores. However, since Cell BLAST only propagates the scores from a children node to its parents, it is not able to annotate unseen cell types that none of its children has been seen in the training data. In contrast, our method is able to do it by using the Cell Ontology graph. Despite the conceptual difference between Cell BLAST and OnClass, we still used Cell BLAST as a comparison approach. Due to the large number of genes that cannot be handled by Cell BLAST using 128 GB RAM, we only considered 10,000 genes that have the largest gene expression variance as features for datasets that have more than 20,000 genes. Although these five methods were able to classify cells into a “unknown” cell type, they were not able to classify these cells into the specific cell type. To enable a fair comparison, we further proposed to extend these three approaches by classifying cells belonging to the unknown cell type to a specific cell type. In particular, when a cell was annotated as the unknown cell type, we first found the seen cell type that had the largest confidence score for this cell. We then annotated the cell to the nearest unseen cell type of this seen cell type on the Cell Ontology graph.

### Cross-dataset prediction

We performed cross-dataset evaluation on 6 datasets, including Muris FACS, Muris droplet, Lemur 1, Lemur 2, Lemur 3, and Lemur 4. We trained on all cells from one dataset and tested on all cells from another dataset. We used the same data preprocessing procedure as ACTINN did^13^. In particular, we only considered genes that were in both the training set and the test set. We then calculated the log count values for the gene expression. Finally, genes with highest 1% expression values and genes with the lowest 1% expression values were then removed. Raw data were used as the input for both the training and the test set. Only genes that appear in both training and test sets were kept during training and prediction. We mapped genes across different species using the homologous gene symbols available from the Ensembl database^55^.

### Pre-trained model

We trained a pre-trained model using Datasets 1–6. In particular, OnClass was run on these 6 dataset separately and then predicted the cell type for a test cell respectively. Each test cell will then have six probability vectors across all cell types. OnClass then averaged these six cell types to obtain the final prediction of the test cell. The expression of the test cell was not used when training the pre-trained model.

To classify cells in the 26-dataset using this pre-trained model, we first used Scanorama to correct batch effects among 26 datasets. For genes that were only included in the training set but not in the test set, we set the expression values of these genes to zero during prediction. Genes that were only included in the test set but not in the training set (i.e., Tabula Muris Senis and Tabula Microcebus) were excluded during prediction.

### Data integration using OnClass

To integrate the 26 datasets, we used OnClass to generate a probability vector for each cell over all cell types in the Cell Ontology. These vectors were first reduced into a low-dimensional space using principal component analysis. The low-dimensional vectors are then used as the input features for UMAP visualization^56^. We used the silhouette coefficient^36^ to evaluate the clustering accuracy for both our method and expression-based integration. We only considered cell types that can be mapped to the Cell Ontology term in the classification evaluation (Fig. 4e). We considered all cell types in the unsupervised evaluation which does not require the cell type to be included in the Cell Ontology (Fig. 5a, b). These 9 cell types are grouped into 6 groups following Scanorama^35^. We have now further explained it in the “Methods” section.

### Marker genes identification

We used Spearman correlation to find marker genes so that all cells could be taken into consideration. We calculated a correlation coefficient between each cell type, including seen cell types and unseen cell types, and each gene. For each cell type, we obtained a vector where each dimension was the probability of a cell being classified to this cell type. For each gene, we obtained a vector where each dimension was the expression of this gene in a cell. The correlation coefficient was then calculated based on these two vectors. We used a correlation coefficient threshold of 0.4 to determine marker genes. The accuracy of the marker genes identified was evaluated using the rank-based metric AUROC without setting a cutoff. The ground truth vector is a binarized vector for each cell type, where 1 means this gene is a known marker and otherwise 0. Curated marker genes of 69 Cell Ontology terms were collected from literature by experts (Supplementary Data 4). 28 cell types in Tabula Muris Senis are in these 69 Cell Ontology terms and thus had curated marker genes that we could use for evaluation purposes. To classify a new cell according to marker genes, we used the mean of the expression of marker genes of each Cell Ontology term as the predicted score for that Cell Ontology term. A larger score indicated that the cell more likely belonged to that Cell Ontology term.

### Statistical analysis

We used the scipy.stats^57^ Python package implementation of the one-sided independent *t*-test, two-sided *t*-test, Spearman correlation statistics, and associated *P*-values used in this study. We used the scikit-learn Python package implementation of one-vs-rest logistic regression, SVM, silhouette coefficients, AUROC, and AUPRC used in this study^58^.

### Reporting summary

Further information on research design is available in the Nature Research Reporting Summary linked to this article.

## Supplementary information

Supplementary Information

Description of Additional Supplementary Files

Supplementary Data 1

Supplementary Data 2

Supplementary Data 3

Supplementary Data 4

Reporting Summary

## Data Availability

All datasets used in this study are available at: https://onclass.readthedocs.io/en/latest/datasets.html. Tabula Muris Senis data is available on GEO (https://www.ncbi.nlm.nih.gov/geo/query/acc.cgi?acc=GSE149590). Lemur data is available on Figshare (https://figshare.com/projects/Tabula_Microcebus/112227). HLCA data is available on Synapse (https://www.synapse.org/#!Synapse:syn21041850). Allen Brain data is available on Allen Brain Atlases data portal (https://portal.brain-map.org/).

## Code availability

OnClass code is available at https://github.com/wangshenguiuc/OnClass and 10.5281/zenodo.5167667. The OnClass server can be found at http://onclass.ds.czbiohub.org/.
