# Supplementary material for: Leveraging the Cell Ontology to classify unseen cell types
Source: Nat Commun. 2021 Sep 21;12:5556. doi: 10.1038/s41467-021-25725-x (PMC8455606; doi:10.1038/s41467-021-25725-x)
Supplement: Supplementary file 1 — Supplementary Information [file 41467_2021_25725_MOESM1_ESM.pdf]

Supplementary Note for "Unifying single-cell annotations based on the Cell Ontology"

Leveraging the Cell Ontology to classify unseen cell types

The Cell Ontology is a directed acyclic graph (DAG) over cell type labels, which are called Cell Ontology terms in this paper. Edges in this graph represent various semantic relationships. In this paper, we only consider the 'is a' relationships, which result in a hierarchy of cell type labels with edges going from general to specific cell type terms. Embedding the Cell Ontology into the low-dimensional space consists of two steps. Firstly, random walk with restart (RWR) is used to calculate a 'diffusion state' vector for each cell type according to its distances to all other cell types. This diffusion state vector has a length equals to the total number of cell types in the Cell Ontology graph. Each bit in this vector represents the RWR-based distance to another cell type on the Cell Ontology graph. Secondly, singular value decomposition (SVD) is used to reduce the dimensionality of these high-dimensional features into a low-dimensional space, encoding the embedding vector of each cell type.

Random walk with restart on the Cell Ontology Graph We first perform random walk with restart on the Cell Ontology graph. RWR is different from conventional random walks in that it introduces a pre-defined probability of restarting at the initial cell type after every iteration.

Formally, let $\mathbf{A}$ denote the adjacency matrix of a cell type graph with n cell types, where each node is a cell type and $\mathbf{A}_{i,j} = 1$ if and only if cell type i is the child or the parent of cell

type j on the Cell Ontology graph. Let $\mathbf{B}_{i,j}$ be the transition matrix of $\mathbf{A}$. Each entry $\mathbf{B}_{i,j}$ in the transition matrix $\mathbf{B}$ represents the probability of a transition from cell type i to cell type j and is defined as:

$$\mathbf{B}_{i,j} = \frac{\mathbf{A}_{i,j}}{\sum_{j'} \mathbf{A}_{i,j'}}. \tag{1}$$

Next, let S_i^t be an n-dimensional distribution vector in which each entry stores the probability of a cell type being visited from cell type i after t steps, RWR from cell type i with restart probability p_r is defined as:

$$S_i^{t+1} = (1 - p_r)S_i^t\mathbf{B} + p_r o_i, \tag{2}$$

where o_i is an n-dimensional distribution vector with $o_i(i) = 1$ and $o_i(j) = 0, \forall i \neq j$. Note that the restart probability controls the relative influence of global and local topological information in the diffusion, where a larger value places greater emphasis on the local structure. We can obtain the stationary distribution S_i^∞ of RWR at the fixed point of this iteration, and we refer to this as the 'diffusion state' S_i of cell type i (i.e. $S_i = S_i^\infty$) , using the same definition as previous work [1]. Intuitively, the jth entry S_{ij} stores the probability that RWR starts at cell type i and ends up at cell type j in equilibrium. The fact that two cell types having similar diffusion states implies they are in similar positions with respect to other cell types in the Cell Ontology graph, which may reflect cell type similarity.

However, the diffusion states are not entirely accurate, partially due to the noisy and incomplete nature of the Cell Ontology. Moreover, high dimensionality imposes additional computational constraints on directly using diffusion states as features for classification or regression

tasks.

Dimensionality reduction on cell type diffusion states To address this issue, OnClass employs the following dimensionality reduction scheme as the previous work [2]. The probability assigned to cell type j in the diffusion state of cell type i is modeled as

$$S_{ij}^{'} = \frac{e^{x_i^T z_j}}{\sum_{j'} e^{x_i^T z_{j'}}}, \tag{3}$$

where $\forall i, z_i, x_i \in R^q$ for $q \ll n$. We refer to z_i as the context feature and x_i as the cell type feature of cell type i both capturing the topological properties of the graph. If x_i and z_j are close in direction and have large inner product, then it is likely that cell type j is frequently visited in the random walk starting from cell type i. OnClass takes a set of observed diffusion states $S = \{S_1, \ldots, S_n\}$ as input and optimizes over z and x for all cell types, using KL-divergence as the objective function.

Instead of using gradient descent to optimize the loss function, we solve this optimization problem by using the classic singular value decomposition [3], which substantially decreases the computation time. To avoid taking a logarithm of zeros, we add a small positive constant to s_{ij} and compute the logarithm diffusion state matrix $\mathbf{L}$ as:

$$\mathbf{L} = \ln(\mathbf{S} + \mathbf{Q}) - \ln(\mathbf{Q}). \tag{4}$$

where $Q \in R^{n \times n}$ with $Q_{ij} = \frac{1}{n}, \forall i, j$. With SVD, we decompose $\mathbf{L}$ into three matrices $\mathbf{U}$, $\mathbf{\Sigma}$ and $\mathbf{V}$:

$$\mathbf{L} = \mathbf{U}\mathbf{\Sigma}\mathbf{V}^T, \tag{5}$$

where $U \in R^{n \times n}$,$V \in R^{n \times n}$, and $\mathbf{\Sigma} \in R^{n \times n}$ is the diagonal singular value matrix. To obtain the low-dimensional vectors z_j and x_i with q dimensions, we simply choose the first q singular vectors U_q, V_q and the first q singular values Σ_d . More precisely, let $\mathbf{X} = \{x_1, \ldots, x_n\}$ denote the low-dimensional vector representation matrix, and $\mathbf{Z} = \{z_1, \ldots, z_n\}$ denote the context feature matrix. $\mathbf{X}$ and $\mathbf{Z}$ can be computed as:

$$\mathbf{X} = \mathbf{U_q}\mathbf{\Sigma_q^{0.5}}, \tag{6}$$

$$\mathbf{Z} = \mathbf{V_q}\mathbf{\Sigma_q^{0.5}}, \tag{7}$$

We use $\mathbf{X} = \{x_1, \ldots, x_q\}$ to denote the low-dimensional vector representation matrix of cell type labels. x_j is the vector for cell type j. Importantly, our representation captures not only single-hop parent-child relationships, but also more global patterns such as long-range sibling relationships on the Cell Ontology graph.

1. Cao, M. *et al.* New directions for diffusion-based network prediction of protein function: incorporating pathways with confidence. *Bioinformatics* **30**, i219–i227 (2014).

2. Wang, S., Cho, H., Zhai, C., Berger, B. & Peng, J. Exploiting ontology graph for predicting sparsely annotated gene function. *Bioinformatics* **31**, i357–64 (2015).

3. Golub, G. H. & Reinsch, C. Singular value decomposition and least squares solutions. *Numerische mathematik* **14**, 403–420 (1970).

Supplementary Fig. 1

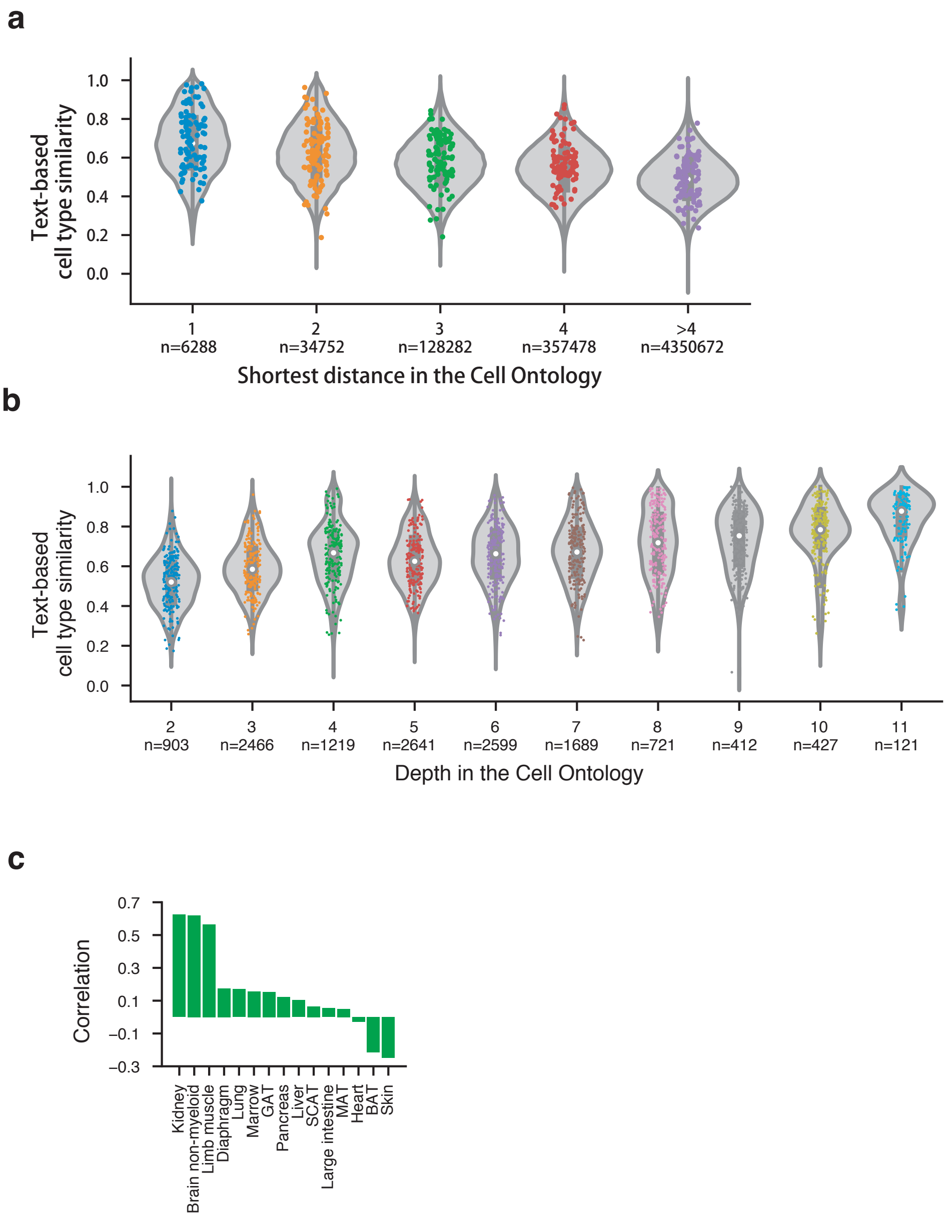

Supplementary Fig. 2

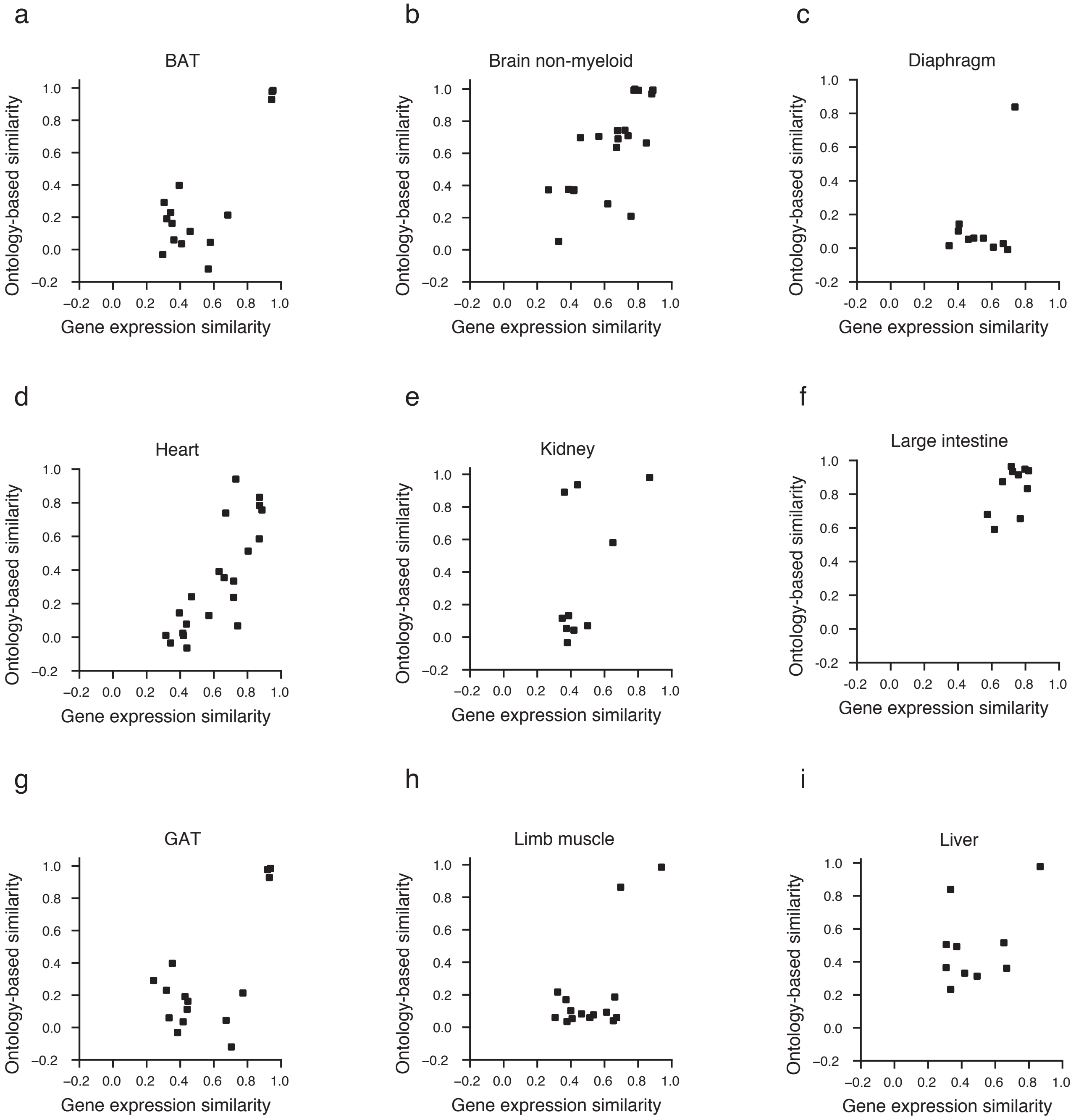

Supplementary Fig. 3

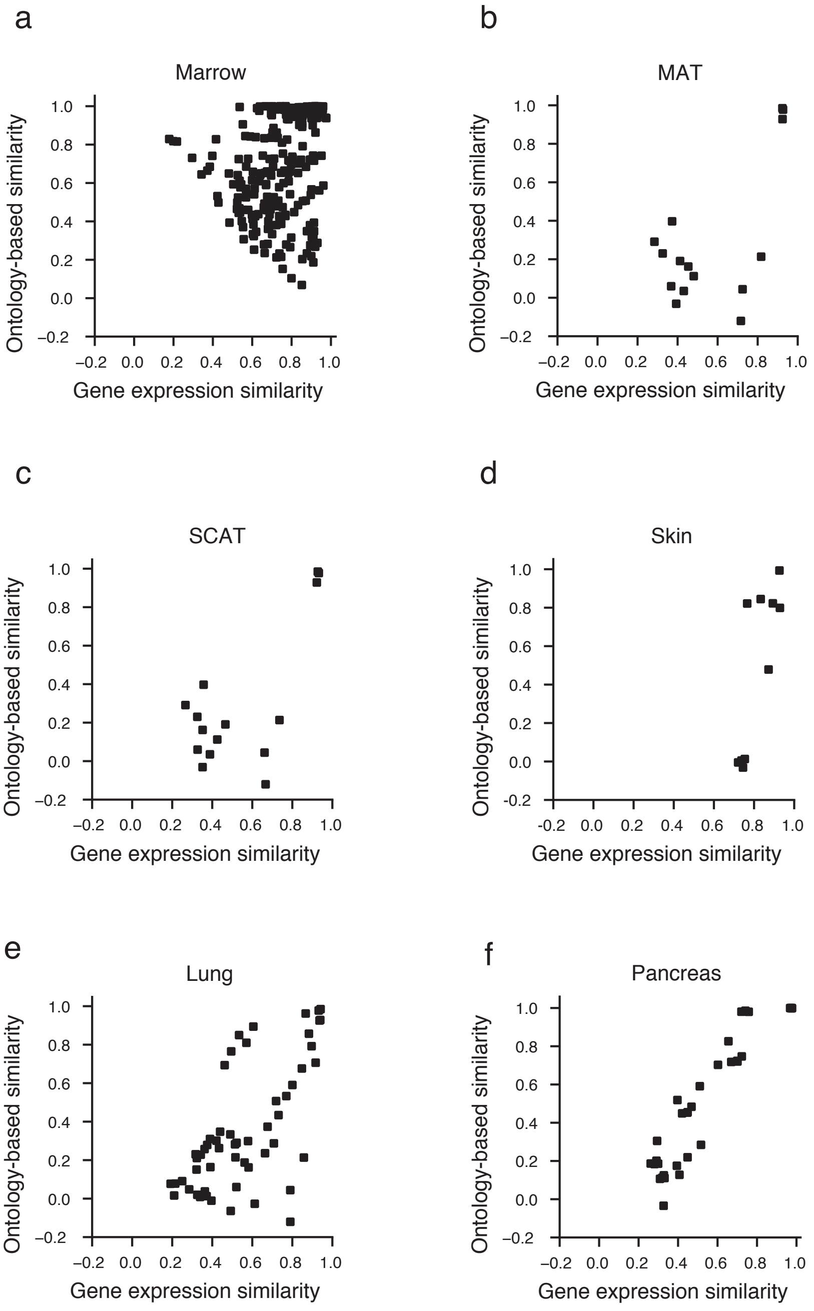

Supplementary Fig. 4

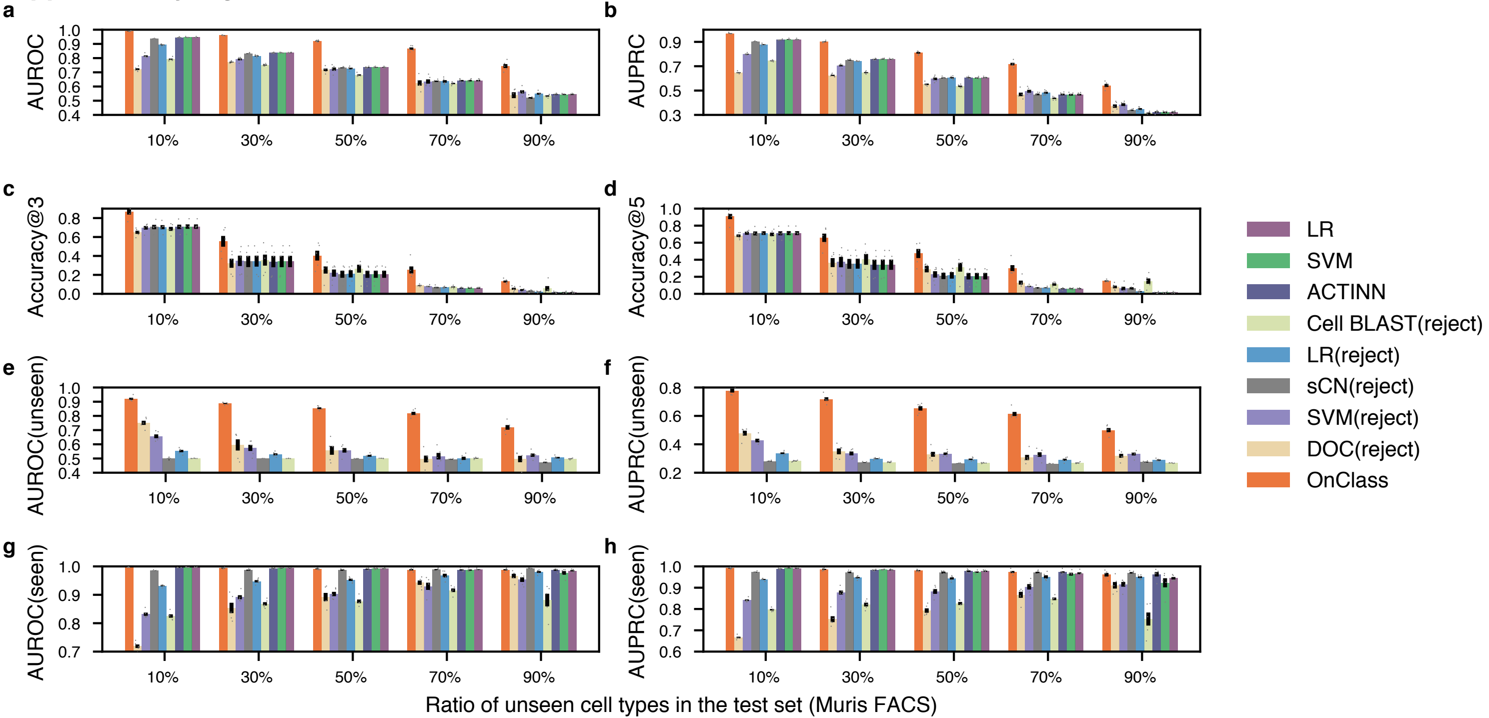

Supplementary Fig. 5

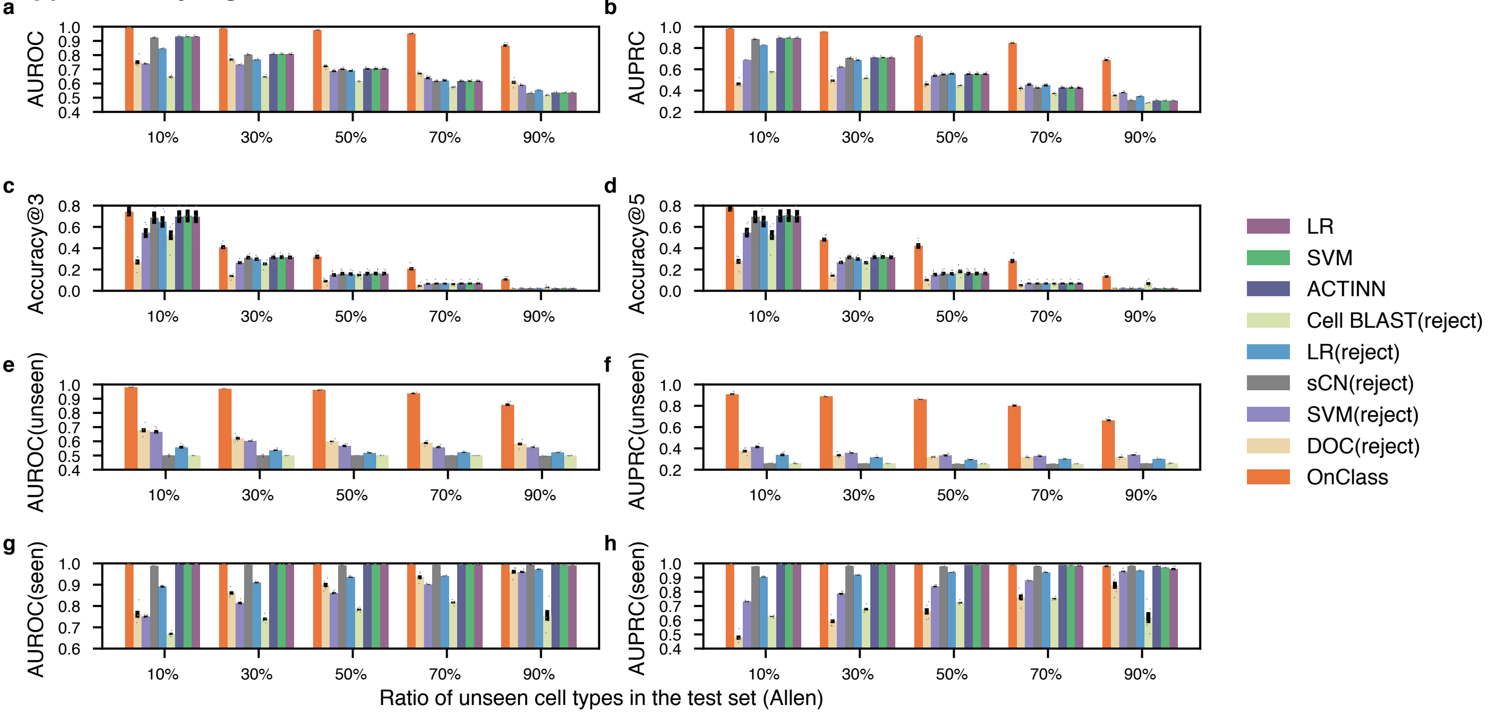

Supplementary Fig. 6

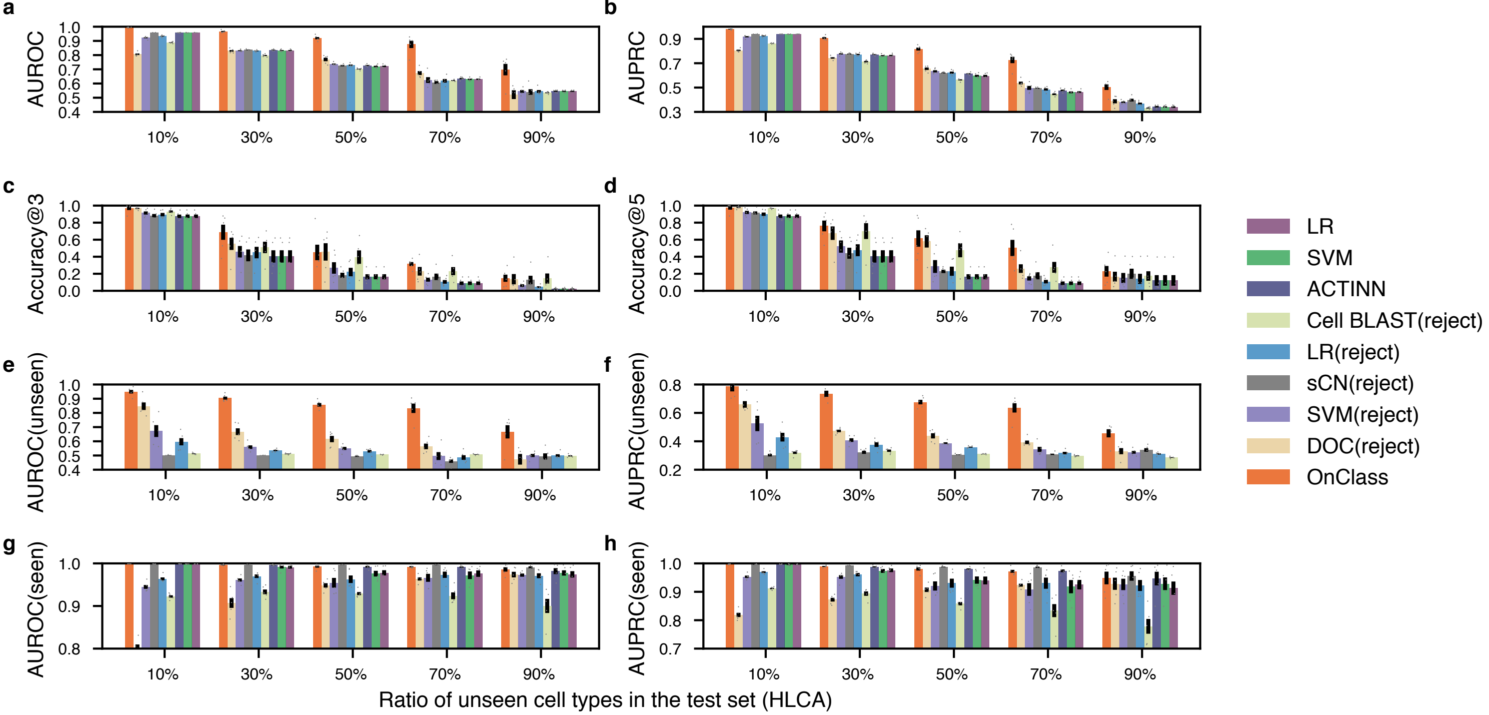

Supplementary Fig. 7

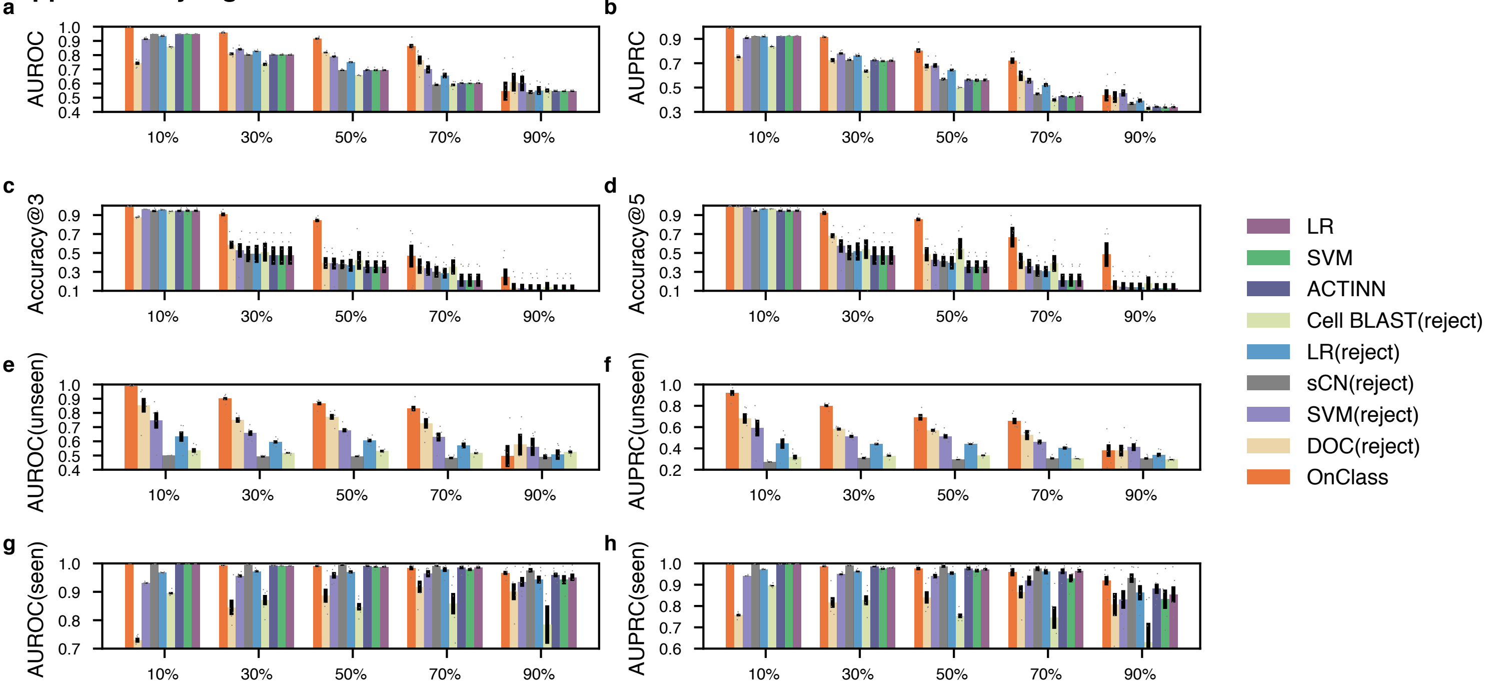

Ratio of unseen cell types in the test set (Lemur 1)

Supplementary Fig. 8

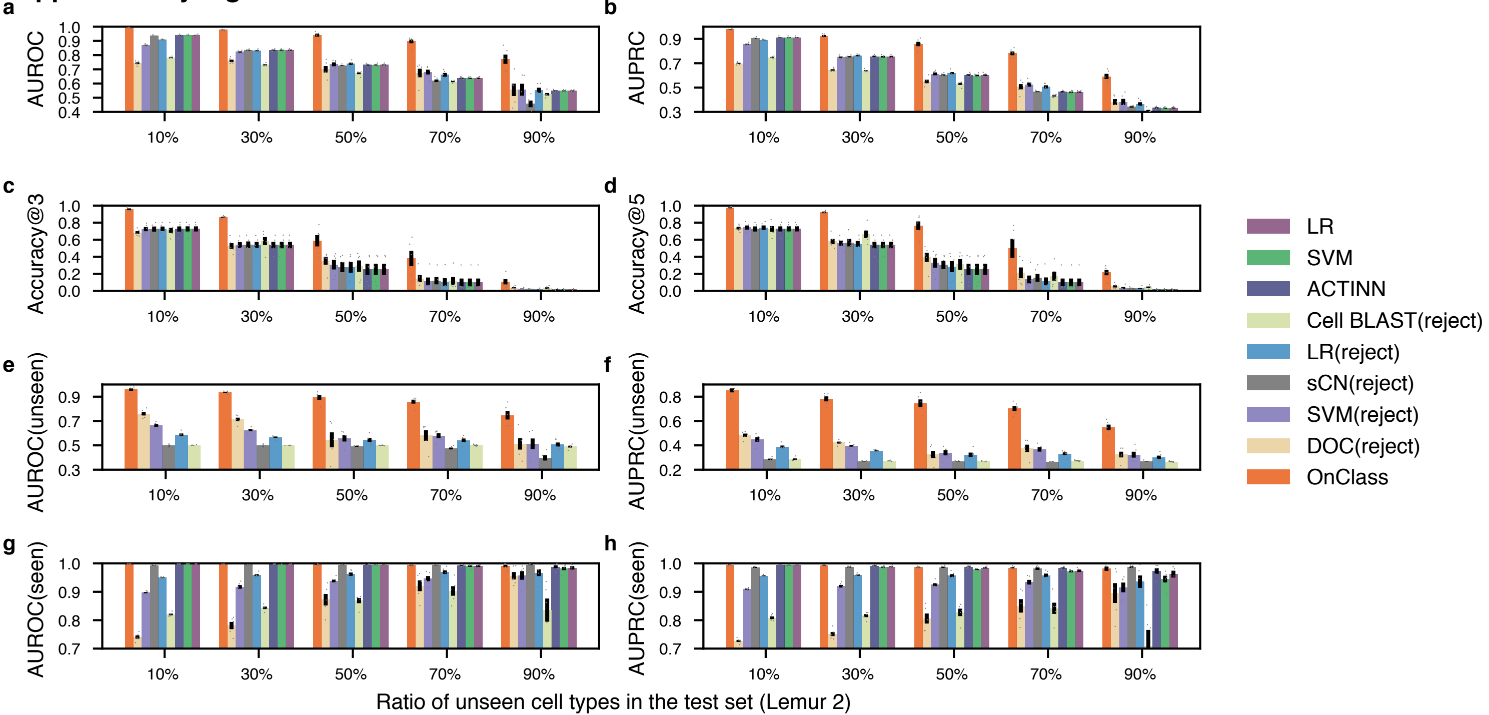

Supplementary Fig. 9

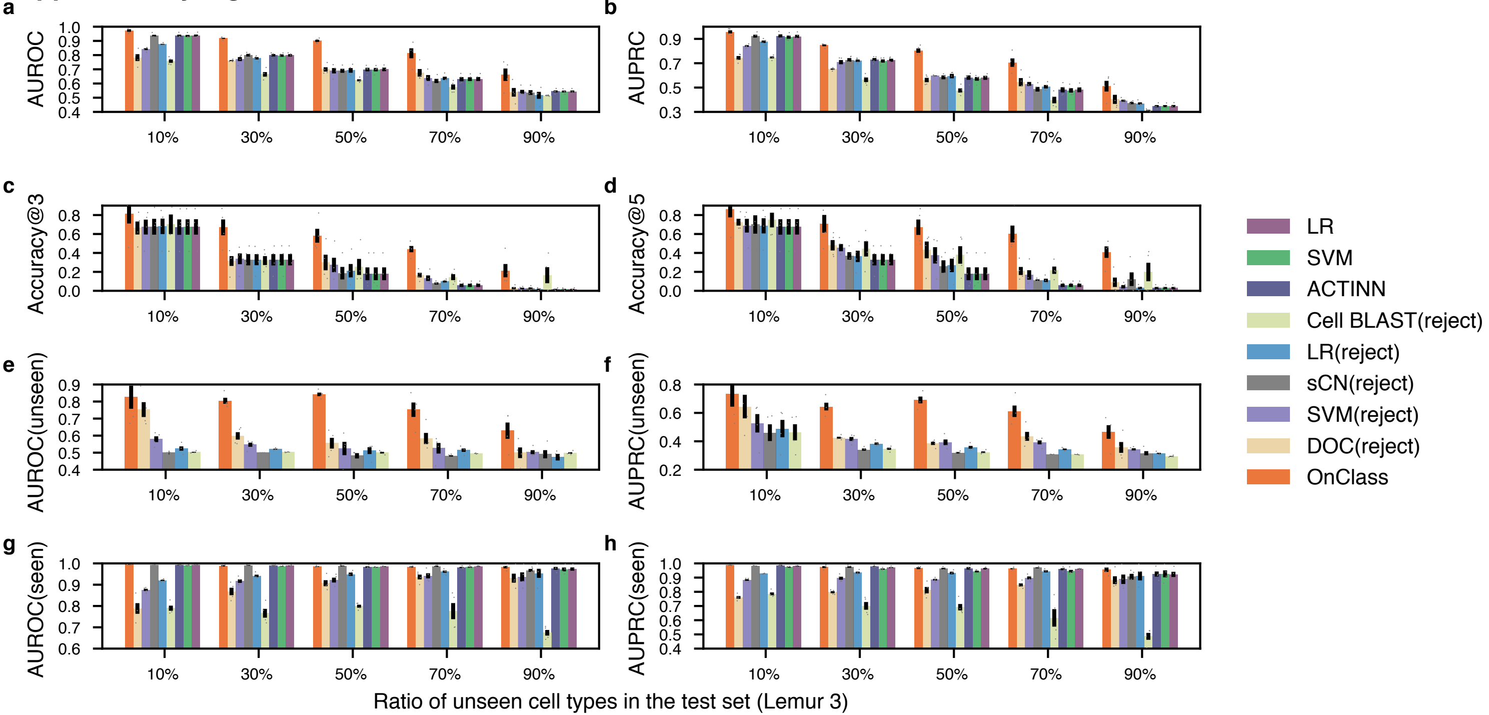

Supplementary Fig. 10

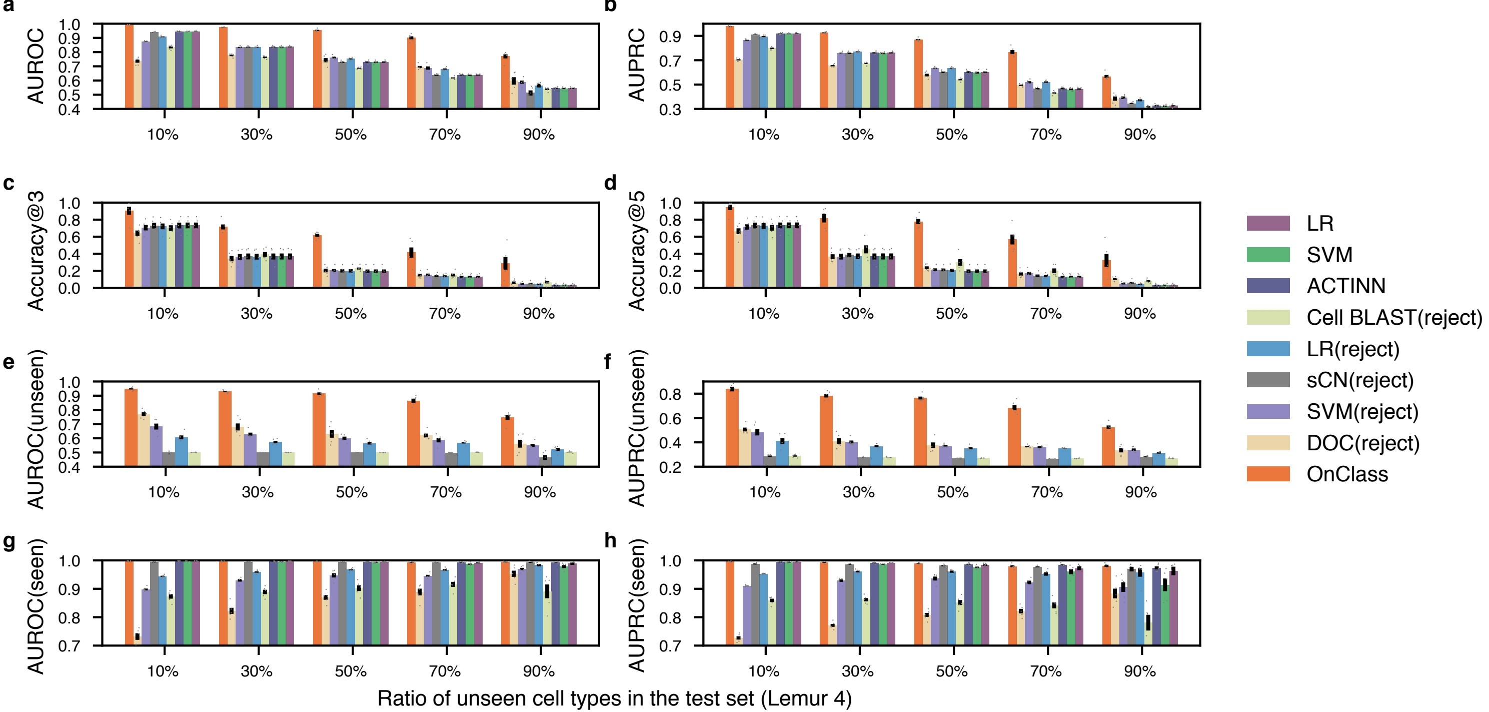

Supplementary Fig. 11

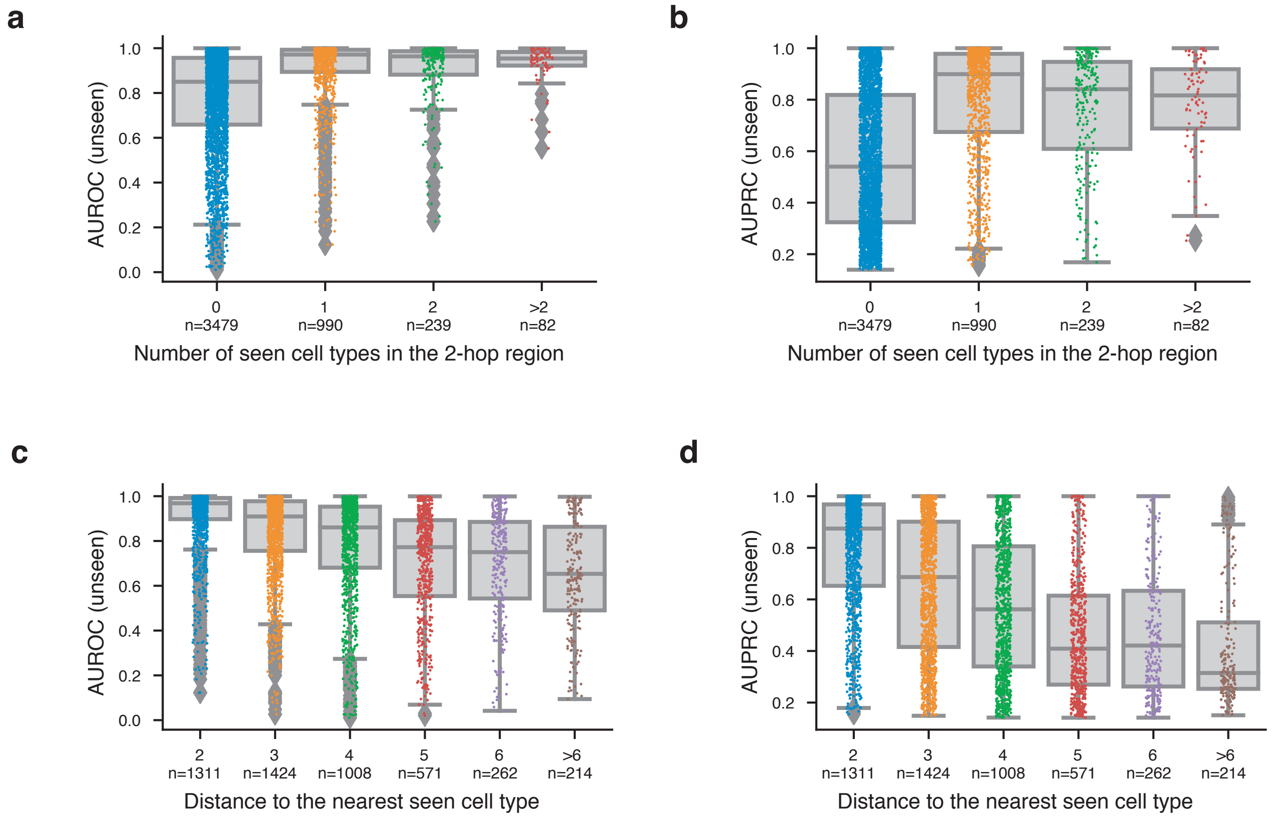

Supplementary Fig. 12

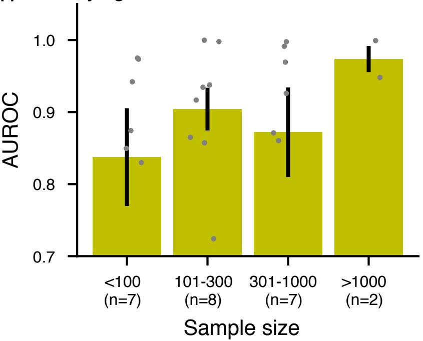

Supplementary Fig. 13

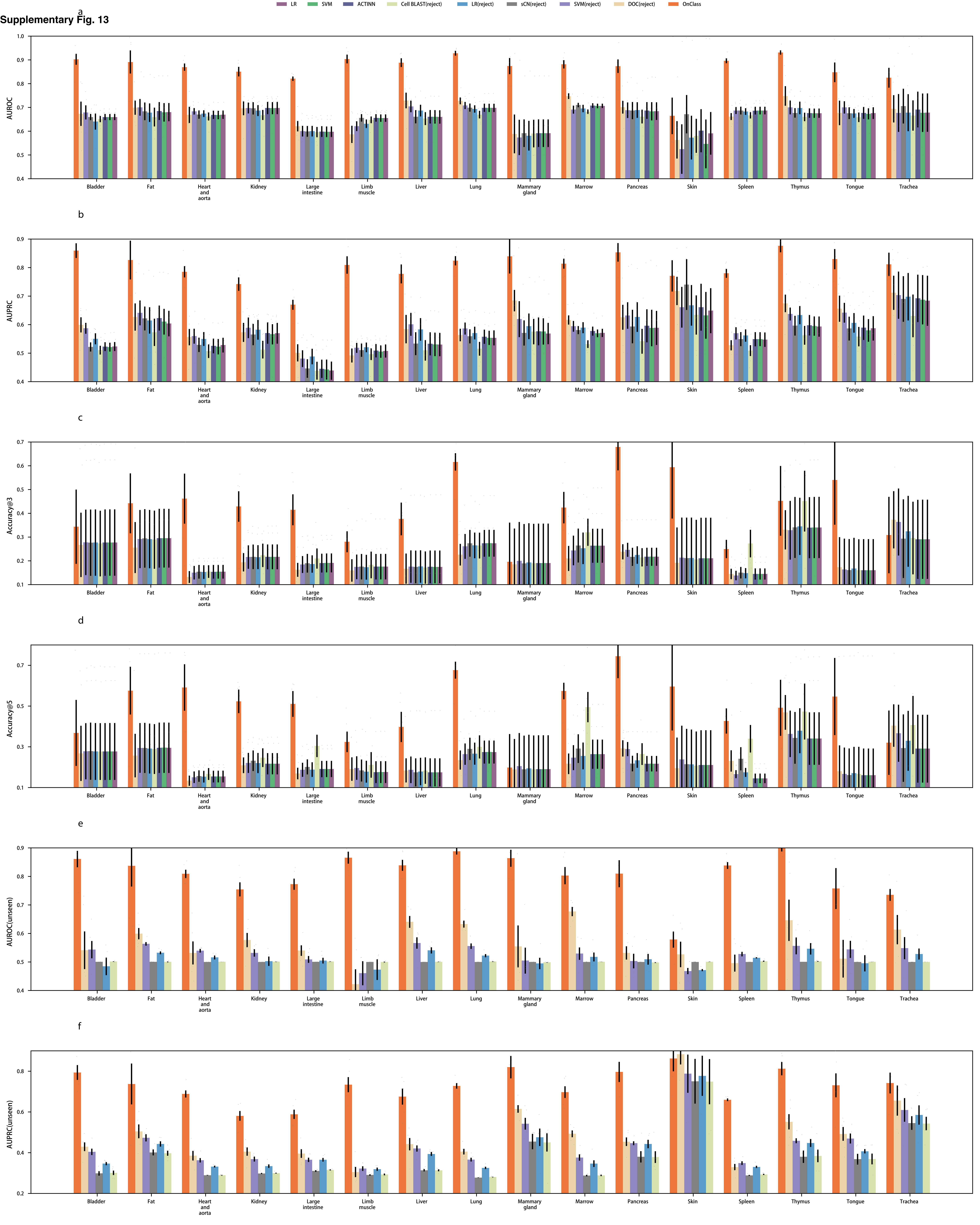

Supplementary Fig. 14

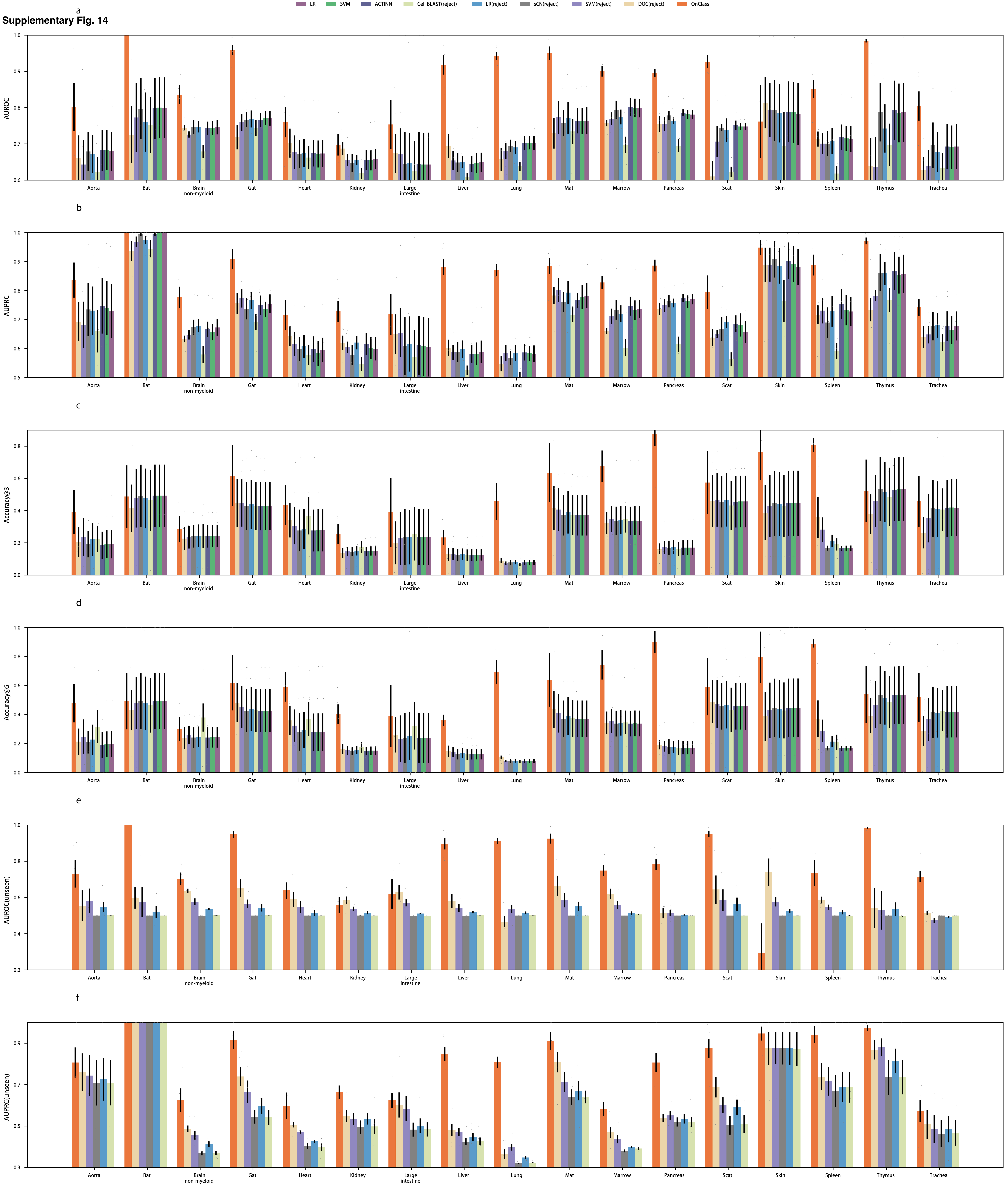

Supplementary Fig. 15

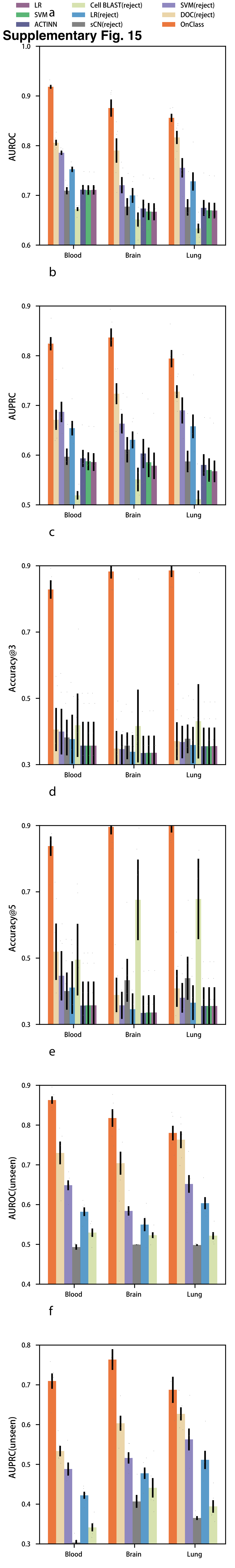

Supplementary Fig. 16

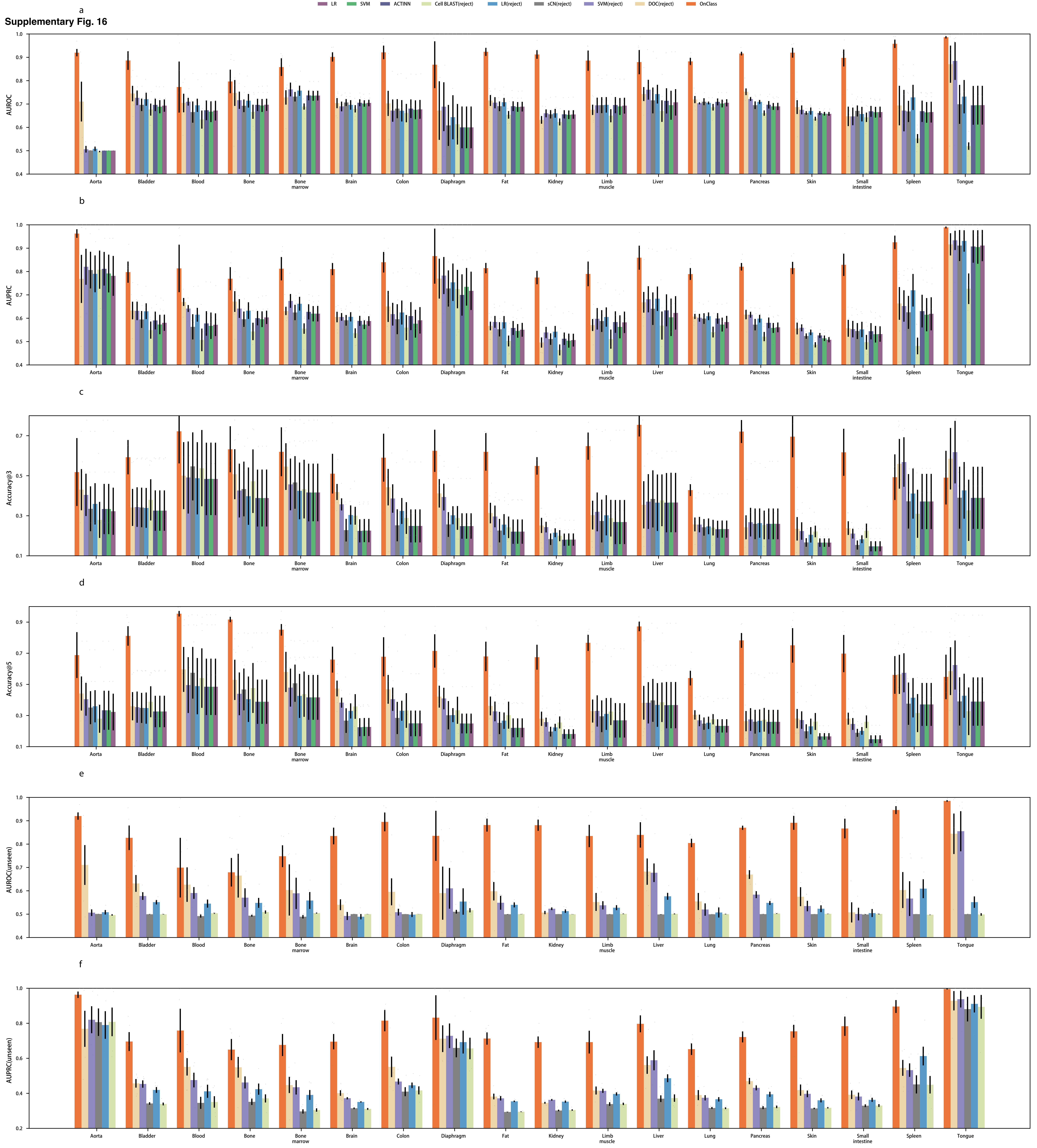

Supplementary Fig. 17

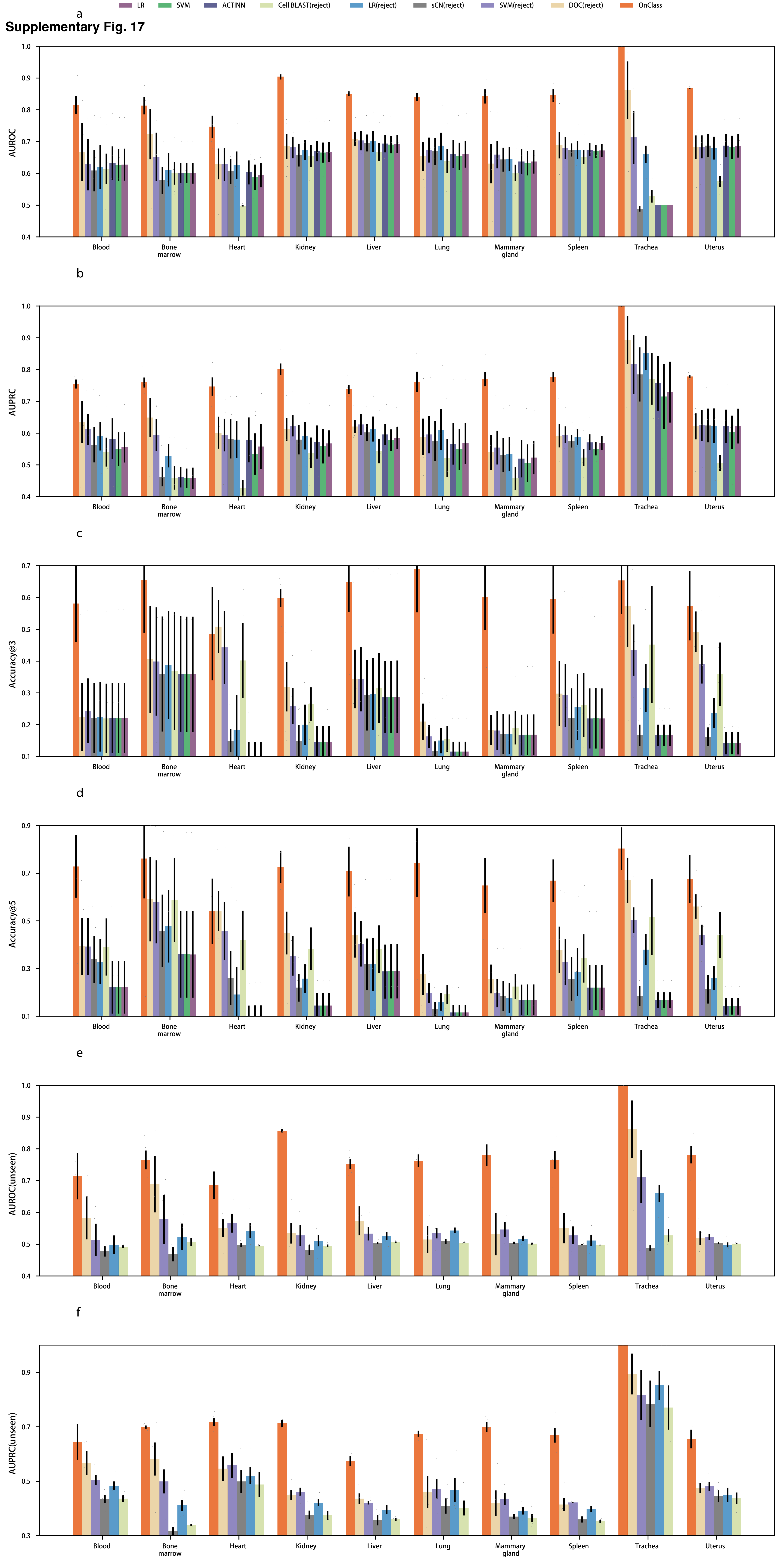

Supplementary Fig. 18

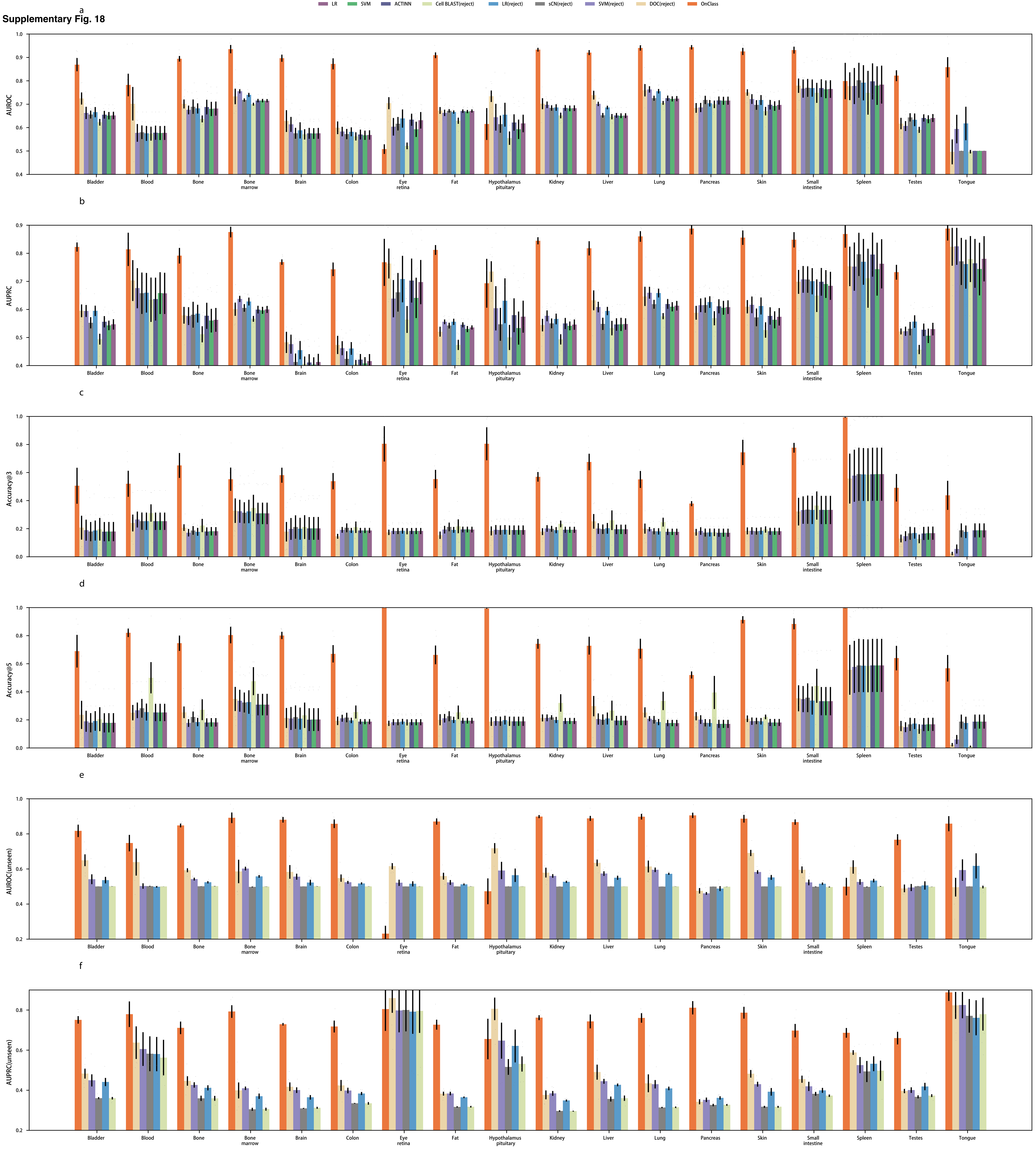

Supplementary Fig. 19

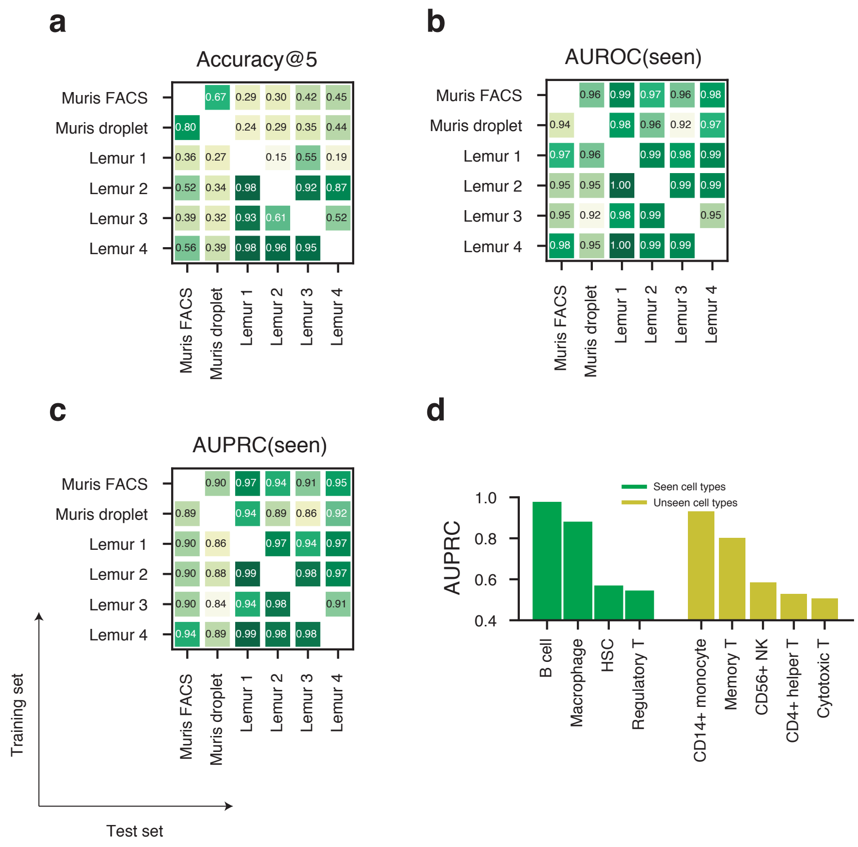

Supplementary Fig. 20

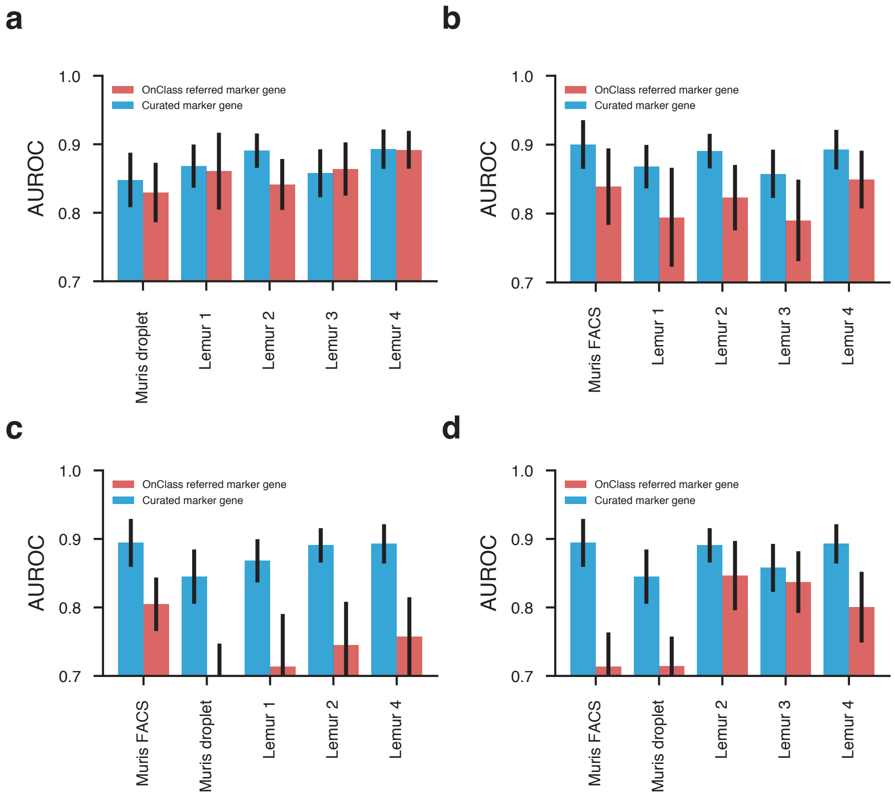

Supplementary Fig. 21

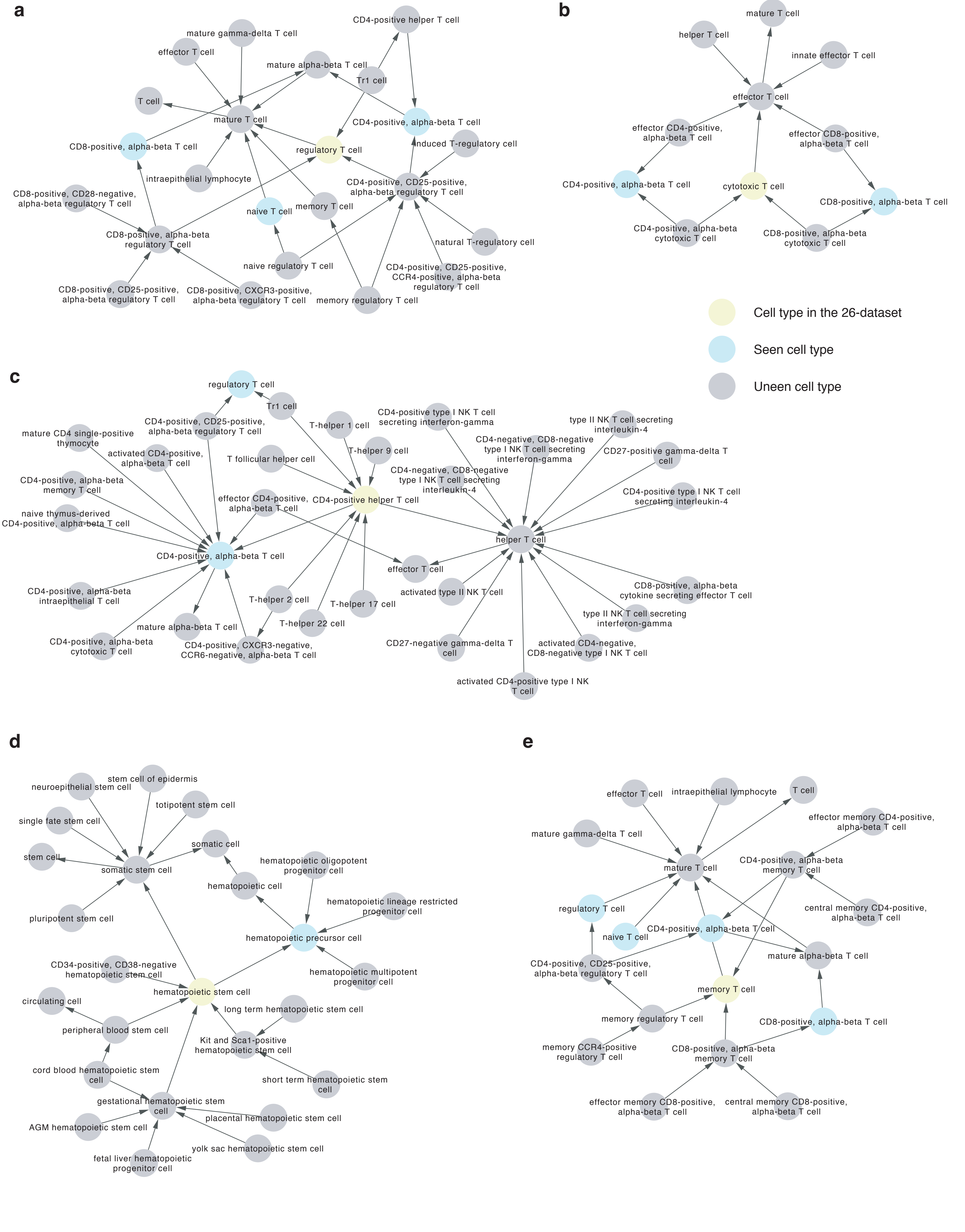

Supplementary Fig. 22

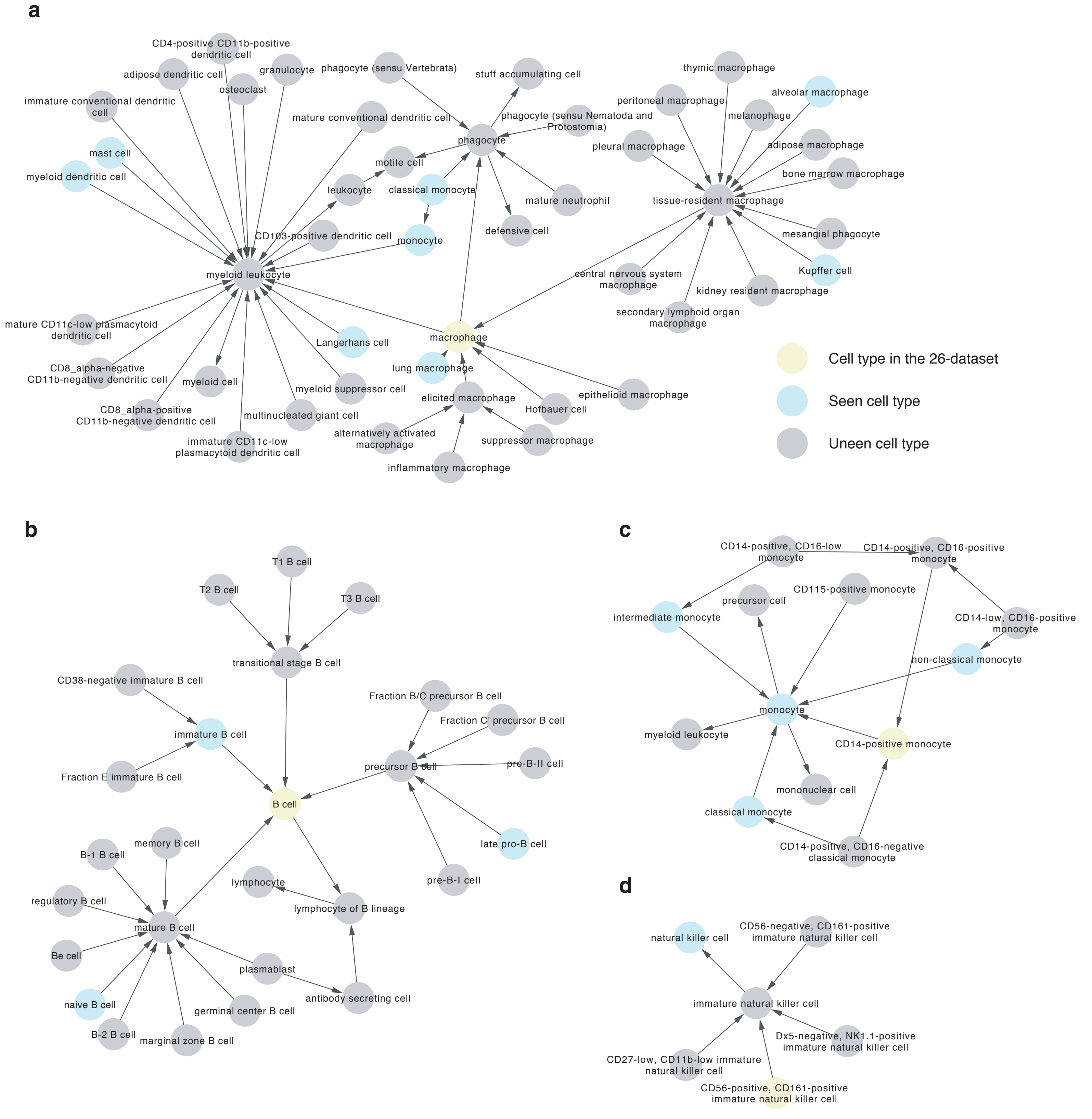

Supplementary Table 1

Cell Ontology 1	Cell Ontology 2	Cell Ontology 2	Cell Ontology class 2
CL:0000099	interneuron	CL:0000534	primary interneuron
CL:0000099	interneuron	CL:0008031	cortical interneuron
CL:0000099	interneuron	CL:0000691	stellate interneuron
CL:0000099	interneuron	CL:0000397	ganglion interneuron
CL:0000099	interneuron	CL:0000246	Mauthner neuron
CL:0000099	interneuron	CL:0000745	retina horizontal cell
CL:0000099	interneuron	CL:0000561	amacrine cell
CL:0000099	interneuron	CL:0011104	interplexiform cell
CL:0000814	mature NK T cell	CL:0000922	type II NK T cell
CL:0000099	interneuron	CL:0000402	CNS interneuron
CL:0000099	interneuron	CL:0000498	inhibitory interneuron
CL:0000099	interneuron	CL:0000103	bipolar neuron
CL:0000814	mature NK T cell	CL:0002127	innate effector T cell
CL:0000814	mature NK T cell	CL:0000921	type I NK T cell
CL:0002393	intermediate monocyte	CL:0001055	CD14-positive, CD16-low monocyte
CL:0000784	plasmacytoid dendritic cell	CL:0001058	plasmacytoid dendritic cell, human
CL:0000784	plasmacytoid dendritic cell	CL:0000991	CD11c-negative plasmacytoid dendritic cell
CL:0000784	plasmacytoid dendritic cell	CL:0000942	thymic plasmacytoid dendritic cell
CL:0000786	plasma cell	CL:0000975	short lived plasma cell
CL:0000784	plasmacytoid dendritic cell	CL:0000989	CD11c-low plasmacytoid dendritic cell
CL:0000098	sensory epithelial cell	CL:1000382	type 2 vestibular sensory cell of stato-acoustic epithelium
CL:0000098	sensory epithelial cell	CL:1000383	type 2 vestibular sensory cell of epithelium of macula of utricle of membranous labyrinth
CL:0000098	sensory epithelial cell	CL:1000384	type 2 vestibular sensory cell of epithelium of macula of saccule of membranous labyrinth
CL:0000098	sensory epithelial cell	CL:1000385	type 2 vestibular sensory cell of epithelium of crista of ampulla of semicircular duct of membranous labyrinth
CL:0002543	vein endothelial cell	CL:2000076	hindlimb stylopod vein endothelial cell
CL:0000098	sensory epithelial cell	CL:1000379	type 1 vestibular sensory cell of epithelium of macula of utricle of membranous labyrinth
CL:0000098	sensory epithelial cell	CL:0002167	olfactory epithelial cell
CL:0000098	sensory epithelial cell	CL:1000380	type 1 vestibular sensory cell of epithelium of macula of saccule of membranous labyrinth
CL:0000098	sensory epithelial cell	CL:1000378	type 1 vestibular sensory cell of stato-acoustic epithelium
CL:0000098	sensory epithelial cell	CL:1000381	type 1 vestibular sensory cell of epithelium of crista of ampulla of semicircular duct of membranous labyrinth

Supplementary Table 2

Dataset	#cells	#genes	Ontology
FACS cells in Tabula Muris Senis (Muris FACS)	73879	22966	Cell Ontology
Droplet cells in Tabula Muris Senis (Muris droplet)	114631	20138	Cell Ontology
Lemur 1 in Tabula Microcebus (Lemur 1)	17614	31509	Cell Ontology
Lemur 2 in Tabula Microcebus (Lemur 2)	76352	31509	Cell Ontology
Lemur 3 in Tabula Microcebus (Lemur 3)	21187	31509	Cell Ontology
Lemur 4 in Tabula Microcebus (Lemur 4)	75501	31509	Cell Ontology
Allen Brain Atlas (Allen)	60351	45768	Allen Ontology
26-dataset	105476	5216	Cell Ontology
HLCA	46576	26485	Cell Ontology

Supplementary Table 3

Cell type name	Cell Ontology ID
B cell	CL:0000236
Macrophage	CL:0000235
HSC	CL:0000037
CD56+ NK	CL:0002338
CD4+ helper T	CL:0000492
Regulatory T	CL:0000815
Cytotoxic T	CL:0000910
CD14+ monocyte	CL:0001054
Memory T	CL:0000813

Supplementary Table 4

Dataset	Cell type in 26-dataset	Nearest seen cell type	Distance
muris_facs	B cell	immature B cell	1
muris_facs	macrophage	lung macrophage	1
muris_facs	hematopoietic stem cell	hematopoietic stem cell	0
muris_facs	CD56-positive, CD161-positive immature natural killer cell	natural killer cell	2
muris_facs	CD4-positive helper T cell	CD4-positive, alpha-beta T cell	1
muris_facs	regulatory T cell	regulatory T cell	0
muris_facs	cytotoxic T cell	CD8-positive, alpha-beta T cell	2
muris_facs	CD14-positive monocyte	intermediate monocyte	2
muris_facs	peripheral blood mononuclear cell	hematopoietic stem cell	3
muris_facs	memory T cell	regulatory T cell	2
muris_droplet	B cell	immature B cell	1
muris_droplet	macrophage	lung macrophage	1
muris_droplet	hematopoietic stem cell	hematopoietic stem cell	0
muris_droplet	CD56-positive, CD161-positive immature natural killer cell	natural killer cell	2
muris_droplet	CD4-positive helper T cell	CD4-positive, alpha-beta T cell	1
muris_droplet	regulatory T cell	regulatory T cell	0
muris_droplet	cytotoxic T cell	CD8-positive, alpha-beta T cell	2
muris_droplet	CD14-positive monocyte	non-classical monocyte	2
muris_droplet	peripheral blood mononuclear cell	hematopoietic stem cell	3
muris_droplet	memory T cell	naive T cell	2

Supplementary Fig. 1. Analysis of the Cell Ontology text description. a, Violin plot showing the text-based cell type similarity of cell types across different shortest distances on the Cell Ontology graph. n represents the number of cell type pairs. Minima, maxima, centre, bounds of box and whiskers represent quantile 1-1.5*interquartile range (IQR), quantile 3+1.5*IQR, median, quantile 1 and quantile 3. **b**, Violin plot showing the text description-based cell type similarity of cell type siblings across different depths on the Cell Ontology graph. n represents the number of cell type pairs. Minima, maxima, centre, bounds of box and whiskers represent quantile 1-1.5*interquartile range (IQR), quantile 3+1.5*IQR, median, quantile 1 and quantile 3. **c,** Bar plot showing the correlation between text-based cell type similarity and gene expression-based cell type similarity.

Supplementary Fig. 2. Comparison between the Cell Ontology graph-based similarity and the gene expression-based similarity on 9 tissues. a-i, Scatter plots showing the correlations between the Cell Ontology-based cell type similarity and the gene expression-based cell type similarity in BAT (P-value = 4e-16, r = 0.80, n = 15) (a), Brain non-myeloid (P-value = 3e-4, r = 0.74, n = 21) (b), Diaphragm (P-value = 3e-1, r = 0.80, n = 15) (c), Heart (P-value = 8e-6, r = 0.81, n = 21) (d), Kidney (P-value = 2e-1, r = 0.53, n = 10) (e), GAT (P-value = 8e-3, r = 0.66, n = 15) (f), Large intestine (P-value = 1e-1, r = 0.56, n = 10) (g), Limb muscle (P-value = 9e-3, r = 0.65, n = 15) (h), and Liver (P-value = 3e-1, r = 0.44, n = 10) (i). Two-sided Pearson correlation P-values are reported here.

Supplementary Fig. 3. Comparison between the Cell Ontology graph-based similarity and the gene expression-based similarity on 6 tissues. a-f, Scatter plots showing the correlations between the Cell Ontology-based cell type similarity and the gene expression-based cell type similarity in Marrow (P-value = 2e-5, r = 0.28, n = 231) (a), MAT (P-value = 2e-2, r = 0.63, n = 21) (b), SCAT (P-value = 4e-3, r = 0.70, n = 15) (c), and Skin (P-value = 5e-3, r = 0.80, n = 10) (d), Lung (P-value = 1e-7, r = 0.65, n = 55) (e) and Pancreas (P-value = 1e-15, r = 0.93, n = 36) (f). Two-sided Pearson correlation P-values are reported here.

Supplementary Fig. 4. Performance of OnClass on unseen cell type annotation in Muris FACS. a-h, Bar plots comparing OnClass and existing methods in terms of AUROC (a), AUPRC (b), Accuracy@3 (c), Accuracy@5 (d), AUROC on unseen cell types (e), AUPRC on unseen cell types (f), AUROC on seen cell types (g), and AUPRC on seen cell types (h). x-axis shows the proportion of unseen cell types in the test data. Error bar represents standard errors across 5 replicates. Mean is used to measure the centre for the error bar.

Supplementary Fig. 5. Performance of OnClass on unseen cell type annotation in Allen Brain Atlas. a-h, Bar plots comparing OnClass and existing methods in terms of AUROC (a), AUPRC (b), Accuracy@3 (c), Accuracy@5 (d), AUROC on unseen cell types (e), AUPRC on unseen cell types (f), AUROC on seen cell types (g), and AUPRC on seen cell types (h). x-axis shows the proportion of unseen cell types in the test data. Error bar represents standard errors across 5 replicates. Mean is used to measure the centre for the error bar.

Supplementary Fig. 6. Performance of OnClass on unseen cell type annotation in Human Lung Cell Atlas (HLCA). a-h, Bar plots comparing OnClass and existing methods in terms of AUROC (a), AUPRC (b), Accuracy@3 (c), Accuracy@5 (d), AUROC on unseen cell types (e), AUPRC on unseen cell types (f), AUROC on seen cell types (g), and AUPRC on seen cell types (h). x-axis shows the proportion of unseen cell types in the test data. Error bar represents standard errors across 5 replicates. Mean is used to measure the centre for the error bar.

Supplementary Fig. 7. Performance of OnClass on unseen cell type annotation in Lemur 1. a-h, Bar plots comparing OnClass and existing methods in terms of AUROC (a), AUPRC (b), Accuracy@3 (c), Accuracy@5 (d), AUROC on unseen cell types (e), AUPRC on unseen cell types (f), AUROC on seen cell types (g), and AUPRC on seen cell types (h). x-axis shows the proportion of unseen cell types in the test data. Error bar represents standard errors across 5 replicates. Mean is used to measure the centre for the error bar.

Supplementary Fig. 8. Performance of OnClass on unseen cell type annotation in Lemur 2. a-h, Bar plots comparing OnClass and existing methods in terms of AUROC (a), AUPRC (b), Accuracy@3 (c), Accuracy@5 (d), AUROC on unseen cell types (e), AUPRC on unseen cell types (f), AUROC on seen cell types (g), and AUPRC on seen cell types (h). x-axis shows the proportion of unseen cell types in the test data. Error bar represents standard errors across 5 replicates. Mean is used to measure the centre for the error bar.

Supplementary Fig. 9. Performance of OnClass on unseen cell type annotation in Lemur 3. a-h, Bar plots comparing OnClass and existing methods in terms of AUROC (a), AUPRC (b), Accuracy@3 (c), Accuracy@5 (d), AUROC on unseen cell types (e), AUPRC on unseen cell types (f), AUROC on seen cell types (g), and AUPRC on seen cell types (h). x-axis shows the proportion of unseen cell types in the test data. Error bar represents standard errors across 5 replicates. Mean is used to measure the centre for the error bar.

Supplementary Fig. 10. Performance of OnClass on unseen cell type annotation in Lemur 4. a-h, Bar plots comparing OnClass and existing methods in terms of AUROC (a), AUPRC (b), Accuracy@3 (c), Accuracy@5 (d), AUROC on unseen cell types (e), AUPRC on unseen cell types (f), AUROC on seen cell types (g), and AUPRC on seen cell types (h). x-axis shows the proportion of unseen cell types in the test data. Error bar represents standard errors across 5 replicates. Mean is used to measure the centre for the error bar.

Supplementary Fig. 11. OnClass has better performance for unseen cell types that are near to seen cell types. a,b,c,d, Boxplot showing AUROC of unseen cell types with different numbers of seen cell types in the 2-hop region using AUROC (a) and AUPRC (b), and different distances to the nearest seen cell type using AUROC (c) and AUPRC (d). n represents the number of cell type pairs. Minima, maxima, centre, bounds of box and whiskers represent quantile 1-1.5*interquartile range (IQR), quantile 3+1.5*IQR, median, quantile 1 and quantile 3.

Supplementary Fig. 12. Performance of OnClass on different sample sizes. Barplot showing AUROC of unseen cell types with different numbers of cells using Muris droplet as the training

set and Muris FACS as the test set. Error bar represents standard errors. Mean is used to measure the centre for the error bar.

Supplementary Fig. 13. Performance of OnClass on unseen cell type annotation in Muris droplet across different tissues. a-f, Bar plots comparing OnClass and existing methods in terms of AUROC (a), AUPRC (b), Accuracy@3 (c), Accuracy@5 (d), AUROC on unseen cell types (e) and AUPRC on unseen cell types (f). x-axis shows the tissue. Error bar represents standard errors across 5 replicates. Mean is used to measure the centre for the error bar.

Supplementary Fig. 14. Performance of OnClass on unseen cell type annotation in Muris FACS across different tissues. a-f, Bar plots comparing OnClass and existing methods in terms of AUROC (a), AUPRC (b), Accuracy@3 (c), Accuracy@5 (d), AUROC on unseen cell types (e) and AUPRC on unseen cell types (f). x-axis shows the tissue. Error bar represents standard errors across 5 replicates. Mean is used to measure the centre for the error bar.

Supplementary Fig. 15. Performance of OnClass on unseen cell type annotation in Lemur 1 across different tissues. a-f, Bar plots comparing OnClass and existing methods in terms of AUROC (a), AUPRC (b), Accuracy@3 (c), Accuracy@5 (d), AUROC on unseen cell types (e), and AUPRC on unseen cell types (f). x-axis shows the tissue. Error bar represents standard errors across 5 replicates. Mean is used to measure the centre for the error bar.

Supplementary Fig. 16. Performance of OnClass on unseen cell type annotation in Lemur 2 across different tissues. a-f, Bar plots comparing OnClass and existing methods in terms of AUROC (a), AUPRC (b), Accuracy@3 (c), Accuracy@5 (d), AUROC on unseen cell types (e), and AUPRC on unseen cell types (f). x-axis shows the tissue. Error bar represents standard errors across 5 replicates. Mean is used to measure the centre for the error bar.

Supplementary Fig. 17. Performance of OnClass on unseen cell type annotation in Lemur 3 across different tissues. a-f, Bar plots comparing OnClass and existing methods in terms of AUROC (a), AUPRC (b), Accuracy@3 (c), Accuracy@5 (d), AUROC on unseen cell types (e), and AUPRC on unseen cell types (f). x-axis shows the tissue. Error bar represents standard errors across 5 replicates. Mean is used to measure the centre for the error bar.

Supplementary Fig. 18. Performance of OnClass on unseen cell type annotation in Lemur 4 across different tissues. a-f, Bar plots comparing OnClass and existing methods in terms of AUROC (a), AUPRC (b), Accuracy@3 (c), Accuracy@5 (d), AUROC on unseen cell types (e), and AUPRC on unseen cell types (f). x-axis shows the tissue. Error bar represents standard errors across 5 replicates. Mean is used to measure the centre for the error bar.

Supplementary Fig. 19. Training with different datasets and proportions of unseen cell types highlights OnClass versatility and accuracy. a-c, Heatmaps showing Accuracy@5 (a), seen AUROC (b), and seen AUPRC (c). **d,** Bar plot showing the AUPRC of OnClass on 9 cell types, including 4 present in the training set (green) and 5 not present in the training set (yellow)

Supplementary Fig. 20. OnClass-computed marker genes can accurately classify cells. a-d, Bar plot comparing the cell type classification performance using curated marker genes and OnClass-computed marker genes obtained from Muris FACS (a), Muris droplet (b), Lemur 3 (c), and Lemur 1 (d). Error bar represents standard errors of n = 17, 21, 13, 22, 13, 22 for Muris FACS, Muris droplet, Lemur 1, Lemur 2, Lemur 3, Lemur 4 respectively. Mean is used to measure the centre for the error bar.

Supplementary Fig. 21. The nearby cell types of each cell type in the 26-dataset. a-e, Cell types that are at most 2-hop away on the Cell Ontology graph to regulatory T cell (a), cytotoxic T cell (b), CD4- positive helper T cell (c), hematopoietic stem cell (d), and memory T cell (e) in the 26-dataset are visualized using Cytoscape[45].

Supplementary Fig. 22. The nearby cell types of each cell type in the 26-dataset. a-d, Cell types that are at most 2-hop away on the Cell Ontology graph to macrophage (a), B cell (b), CD14-positive monocyte (c), and CD56-positive, CD161-positive immature natural killer cell (d) in the 26-dataset are visualized using Cytoscape.

Supplementary Table 1. New cell populations suggested by OnClass. Two closest cell Ontology terms are included.

Supplementary Table 2. Summary of the datasets evaluated by OnClass.

Supplementary Table 3. Mapping of the cell types in the 26-dataset to Cell Ontology terms.

Supplementary Table 4. The nearest seen Cell Ontology terms for cell types in the 26-dataset. Distance on the Cell Ontology graph is included.
