# Supplementary material for: Leveraging the Cell Ontology to classify unseen cell types
Source: Nat Commun. 2021 Sep 21;12:5556. doi: 10.1038/s41467-021-25725-x (PMC8455606; doi:10.1038/s41467-021-25725-x)
Supplement: Supplementary file 2 — Description of Additional Supplementary Files [file 41467_2021_25725_MOESM2_ESM.pdf]

Supplementary Data 1. Mapping of free text annotation to the most similar Cell Ontology term using natural language processing approach. The text-based cosine similarity between the free text annotation and the most similar Cell Ontology term and the rank of the correct Cell Ontology term are included.

Supplementary Data 2. Performance of OnClass and comparison approaches on cross dataset prediction.

Supplementary Data 3. Provisional Cell Ontology by including OnClass-computed marker genes to the Cell Ontology. Marker genes are in the "OnClass-computed marker genes" field and sorted in descending order of confidences.

Supplementary Data 4. Marker genes curated by the experts of Tabula Muris Consortium.
