# Supplementary material for: Leveraging the Cell Ontology to classify unseen cell types
Source: Nat Commun. 2021 Sep 21;12:5556. doi: 10.1038/s41467-021-25725-x (PMC8455606; doi:10.1038/s41467-021-25725-x)
Supplement: Supplementary file 7 — Reporting Summary [file 41467_2021_25725_MOESM7_ESM.pdf]

nature research

Corresponding author(s): Russ B. Altman

Last updated by author(s): Aug 5, 2021

Reporting Summary

Nature Research wishes to improve the reproducibility of the work that we publish. This form provides structure for consistency and transparency in reporting. For further information on Nature Research policies, see our Editorial Policies and the Editorial Policy Checklist.

Statistics

For all statistical analyses, confirm that the following items are present in the figure legend, table legend, main text, or Methods section.

n/a	Confirmed	
☐	☒	The exact sample size (n) for each experimental group/condition, given as a discrete number and unit of measurement
☐	☒	A statement on whether measurements were taken from distinct samples or whether the same sample was measured repeatedly
☐	☒	The statistical test(s) used AND whether they are one- or two-sided<br>*Only common tests should be described solely by name; describe more complex techniques in the Methods section.*
☒	☐	A description of all covariates tested
☐	☒	A description of any assumptions or corrections, such as tests of normality and adjustment for multiple comparisons
☐	☒	A full description of the statistical parameters including central tendency (e.g. means) or other basic estimates (e.g. regression coefficient) AND variation (e.g. standard deviation) or associated estimates of uncertainty (e.g. confidence intervals)
☐	☒	For null hypothesis testing, the test statistic (e.g. F, t, r) with confidence intervals, effect sizes, degrees of freedom and P value noted<br>*Give P values as exact values whenever suitable.*
☒	☐	For Bayesian analysis, information on the choice of priors and Markov chain Monte Carlo settings
☒	☐	For hierarchical and complex designs, identification of the appropriate level for tests and full reporting of outcomes
☒	☐	Estimates of effect sizes (e.g. Cohen's d, Pearson's r), indicating how they were calculated

Our web collection on statistics for biologists contains articles on many of the points above.

Software and code

Policy information about availability of computer code

Data collection	N. A.
Data analysis	We used the scipy.stats Python package (v1.5.4) implementation of the one-sided independent t-test, Pearson correlation statistics, Spearman correlation statistics, and associated p-values used in this study. We used the scikit-learn Python package (v0.24.2) implementation of one-vs-rest logistic regression, silhouette coefficients, AUROC, and cohen's kappa statistics used in this study. We used Cytoscape v3.8.2 to plot the Cell Ontology subgraph.

For manuscripts utilizing custom algorithms or software that are central to the research but not yet described in published literature, software must be made available to editors and reviewers. We strongly encourage code deposition in a community repository (e.g. GitHub). See the Nature Research guidelines for submitting code & software for further information.

Data

Policy information about availability of data

All manuscripts must include a data availability statement. This statement should provide the following information, where applicable:

- Accession codes, unique identifiers, or web links for publicly available datasets
- A list of figures that have associated raw data
- A description of any restrictions on data availability

All datasets used in this study are available at:
https://onclass.readthedocs.io/en/latest/datasets.html. Tabula Muris Senis data is available on GEO (GSE149590). Lemur data is available on Figshare (https://figshare.com/projects/Tabula_Microcebus/112227). HLCA data is available on Synapse (https://www.synapse.org/#!Synapse:syn21041850). Allen Brain data is available on Allen Brain Atlases data portal (https://portal.brain-map.org/).

Field-specific reporting

Please select the one below that is the best fit for your research. If you are not sure, read the appropriate sections before making your selection.

[x] Life sciences [ ] Behavioural & social sciences [ ] Ecological, evolutionary & environmental sciences

For a reference copy of the document with all sections, see nature.com/documents/nr-reporting-summary-flat.pdf

Life sciences study design

All studies must disclose on these points even when the disclosure is negative.

Sample size	Sample size is determined by cross-validation.
Data exclusions	No data were excluded.
Replication	We performed cross-validation and averaged results from different folds to simulate replication. Replicates of 5 are used to determine statistical significance.
Randomization	Randomization is performed in cross-validation. Samples are all randomly allocated.
Blinding	Test datasets are not held out during training the machine learning model.

Reporting for specific materials, systems and methods

We require information from authors about some types of materials, experimental systems and methods used in many studies. Here, indicate whether each material, system or method listed is relevant to your study. If you are not sure if a list item applies to your research, read the appropriate section before selecting a response.

Materials & experimental systems

n/a	Involved in the study	
[x]	[ ]	Antibodies
[x]	[ ]	Eukaryotic cell lines
[x]	[ ]	Palaeontology and archaeology
[x]	[ ]	Animals and other organisms
[x]	[ ]	Human research participants
[x]	[ ]	Clinical data
[x]	[ ]	Dual use research of concern

Methods

n/a	Involved in the study	
[x]	[ ]	ChIP-seq
[x]	[ ]	Flow cytometry
[x]	[ ]	MRI-based neuroimaging
